# sTNFRII-Fc modification protects human UC-MSCs against apoptosis/autophagy induced by TNF-α and enhances their efficacy in alleviating inflammatory arthritis

**DOI:** 10.1186/s13287-021-02602-4

**Published:** 2021-10-09

**Authors:** Yingjie Zhao, Xuezhi Yang, Siyu Li, Bingjie Zhang, Susu Li, Xinwei Wang, Yueye Wang, Chengyan Jia, Yan Chang, Wei Wei

**Affiliations:** 1 Key Laboratory of Anti-Inflammatory and Immune Medicine, Anhui Collaborative Innovation Center of Anti-Inflammatory and Immune Medicine, Institute of Clinical Pharmacology, Anhui Medical University, Ministry of Education, Hefei, 230032 China; 2 Department of Clinical Pharmacology, The Second Hospital of Anhui Medical University, Hefei, 230601 China

**Keywords:** Genetic modification, Mesenchymal stem cells, Rheumatoid arthritis, sTNFRII-Fc, TNF-α, Apoptosis, Autophagy

## Abstract

**Background:**

Tumor necrosis factor (TNF)-α inhibitors represented by Etanercept (a fusion protein containing soluble TNF receptor II (sTNFRII) and the Fc segment of human IgG1) play a pivotal role in Rheumatoid arthritis (RA) treatment. However, long-term use increases the risk of infection and tumors for their systemic inhibition of TNF-α, which disrupts the regular physiological function of this molecular. Mesenchymal stem cells (MSCs)-based delivery system provides new options for RA treatment with their “homing” and immune-regulation capacities, whereas inflammatory environment (especially TNF-α) is not conducive to MSCs' therapeutic effects by inducing apoptosis/autophagy. Here, we constructed a strain of sTNFRII-Fc-expressing MSCs (sTNFRII-MSC), aiming to offset the deficiency of those two interventions.

**Methods:**

Constructed sTNFRII-Fc lentiviral vector was used to infect human umbilical cord-derived MSCs, and sTNFRII-MSC stable cell line was generated by monoclonal cultivation. In vitro and vivo characteristics of sTNFRII-MSC were assessed by coculture assay and an acute inflammatory model in NOD/SCID mice. The sTNFRII-MSC were transplanted into CIA model, pathological and immunological indicators were detected to evaluate the therapeutic effects of sTNFRII-MSC. The distribution of sTNFRII-MSC was determined by immunofluorescence assay. Apoptosis and autophagy were analyzed by flow cytometry, western blot and immunofluorescence.

**Results:**

sTNFRII-Fc secreted by sTNFRII-MSC present biological activity both in vitro and vivo. sTNFRII-MSC transplantation effectively alleviates mice collagen-induced arthritis (CIA) via migrating to affected area, protecting articular cartilage destruction, modulating immune balance and sTNFRII-MSC showed prolonged internal retention via resisting apoptosis/autophagy induced by TNF-α.

**Conclusion:**

sTNFRII-Fc modification protects MSCs against apoptosis/autophagy induced by TNF-α, in addition to releasing sTNFRII-Fc neutralizing TNF-α to block relevant immune-inflammation cascade, and thus exert better therapeutic effects in alleviating inflammatory arthritis.

**Supplementary Information:**

The online version contains supplementary material available at 10.1186/s13287-021-02602-4.

## Background

Rheumatoid arthritis (RA) is a chronic inflammatory joint disease of autoimmune nature characterized by pain, swelling, stiffness, and peripheral joint destruction that can severely impair physical function and quality of life, and up to now, there are no satisfactory therapeutic strategies. Although the pathophysiology of RA has not been fully illuminated, the generalized inflammation has been proved to be responsible for the activation of pathogenic cell populations and subsequent synovium hyperplasia and cartilage destruction [1]. In RA, the inflammatory milieu is regulated by a complex network of cytokines, chemokines, proteinases and other bioactivators, and among which tumor necrosis factor (TNF)-α is considered to be the central mediator and upstream molecule involved in the RA immune-inflammatory cascade [2, 3].

TNF-α is originally produced as a trimeric type II transmembrane protein, which can be cleaved to a soluble form by the metalloproteinase TNF-converting enzyme, and primarily secreted by lymphocytes, monocytes and macrophages [4, 5]. This extracellular TNF-α binds to its two receptors, tumor necrosis factor receptor (TNFR) I (expressed on almost all mammalian cell type) and TNFR II (restricted to immune cells and endothelial cells) on the cell membrane, and eventually activates the transcription factor nuclear factor-κB to mediate different physiological processes [6]. In RA condition, TNF-α levels in plasma and synovial fluid were abnormally elevated, which correlated with the degree of disease activities [5, 7]. The overflowing TNF-α mediated the proliferation, activation, differentiation of pathogenic cell subsets and production of autoantibodies, chemokines, pro-inflammatory cytokines (such as interleukin (IL)-1, IL-6 and IL-8) and matrix degrading enzyme (such as matrix metalloproteinase (MMPs) and a disintegrin and metalloproteinase with thrombospondin motifs (ADAMTS)), leading to immune cells infiltration and cartilage/bone destruction [1, 2, 8]. Therefore, the availability and approval of TNF-α-blocking biological agents for use in clinic has revolutionized the treatment of rheumatic disease. TNF inhibitors, represented by Etanercept have achieved great success in the treatment of RA [5, 9]. Etanercept (sTNFRII-Fc) is a synthetic fusion protein, that comprises soluble TNFR II (sTNFRII) and Fc domain of human Immunoglobulin (Ig) G1, which achieves therapeutic effects via competitive binding of both soluble and membrane‐attached TNF-α to impede the activation of downstream signaling [9, 10]. Despite aforesaid success, however, the current therapeutic paradigm of global TNF-α blockade has several limitations, including adverse effects (serious infection), low rates of disease remission and generation of anti-drug antibodies [11, 12]. These limitations were due to systemic delivery of those TNF-α inhibitors, which required higher dosage to treat affected arthritic joints in addition to generating immunogenicity and affecting the inherent physiological function of TNF-α within the hematopoietic lineage [13, 14]. Thus, there is an urgent need for a safe, effective and easily accessible carrier to deliver drugs to the affected areas, and thus reduce the occurrence of adverse reactions.

Mesenchymal stem cells (MSCs), particularly umbilical cord-derived mesenchymal stem cells (UC-MSCs), have been proved to be a good candidate for treatment of autoimmune diseases both in preclinical and clinical studies due to their immune-modulation, anti-inflammation and tissue repair functions [15–17]. In current studies, MSCs were used as targeted drug delivery vehicle due to the following properties: (1) low immunogenicity; (2) ease of isolation and expansion in vitro; (3) established gene transfer systems for MSCs recombination; (4) ability to homing to inflammation and lesion vicinity [18]. However, despite above superiorities, as a cell-based therapeutic strategy, MSCs were sensitive to environmental issues. It is convinced that inflammatory environment (especially TNF-α) is not conducive to the survival and therapeutic effects of MSCs via inducing apoptosis/autophagy [19, 20], impairing immune-modulatory capacity [21, 22], restraining self-renewal [23], inhibiting chondrogenic, adipogenic and osteogenic differentiation [13, 24, 25], increasing immunogenicity and risk of tumorigenesis [23, 26]. Moreover, depletion of TNF-α rescues the therapeutic effects of MSCs [19, 22, 23, 25]. Based on the above, we hypothesized that the genetically-engineered human UC-MSCs overexpressing sTNFRII-Fc fusion protein (sTNFRII-MSC) may have greater effects on alleviating RA. sTNFRII-MSC, on the one hand, migrate to affected tissues and exert immune regulation and tissue repair ability; on the other hand, they regulate local inflammatory microenvironment by secreting sTNFRII-Fc, neutralizing abnormal elevated TNF-α, and improve their own survival by inhibiting apoptosis, autophagy and immunogenicity.

In this study, we attempted to generate a strain of sTNFRII-Fc fusion protein modified human UC-MSCs named sTNFRII-MSC using lentiviral vector. sTNFRII-Fc modification did not alter MSCs’ inherent biological characteristics, meanwhile they secreted high levels of bioactive sTNFRII-Fc which neutralizing TNF-α both in vitro and vivo. Systemic transplantation of these sTNFRII-MSC exhibited stronger effects on alleviating mice collagen induced arthritis (CIA) via restoring immune balance, modulating inflammation and repairing affected joints compared with naïve MSCs and Etanercept. As for the mechanism underlying the better therapeutic effects granted by sTNFRII-MSC, we focus on the apoptosis and autophagy of implanted MSCs induced by TNF-α. sTNFRII-MSC maintained longer in CIA mice than naïve MSCs which was related to the protective effect of sTNFRII-Fc modification against apoptosis and autophagy induced by TNF-α on MSCs. In addition, sTNFRII-MSC exhibited lower immunogenicity and stronger chondrogenic capacity than naïve MSCs in the inflammatory environment. This study constructed a multi-target cellular immunotherapy, which MSCs was used as a vector to deliver sTNFRII-Fc locally as well as exert their own immune-modulatory and tissue-repairing capacity, which provided new therapeutic measures and experimental basis for clinical treatment of autoimmune diseases such as RA.

## Materials and methods

### Cell culture

Frozen human UC-MSCs from healthy donors at passage 3 were provided by Konya biotechnology Co., Ltd (Nanjing, China) and maintained in Dulbecco’s modified Eagle’s medium (DMEM)/F12 (BI, Cromwell, CT, USA) supplied with 10% fetal bovine serum (FBS) (Hyclone, Thermo Fisher Scientific, Waltham, MA, USA), 100 µg/mL streptomycin/penicillin and 2 mM L-glutamine (BI, Cromwell, CT, USA) at 37 ℃ and 5% CO_2_.

Human fibroblast-like synoviocytes (FLS) were isolated from synovial tissue of RA patients after obtaining written informed consent as previously described [27], which was approved by the biomedical ethics committee of Anhui Medical University. Hyperplastic synovium biopsies from RA patients were washed and minced in DMEM (BI, Cromwell, CT, USA) with penicillin/streptomycin. Then, dissected synovial tissues were digested with 0.1 mg/mL of deoxyribonuclease I (Sigma) and 1 mg/mL of collagenase IV (Sigma) and incubated at 37 ℃ for 2 h. Single cells were obtained after filtering and washing, then cultured in DMEM with 20% FBS and 100 µg/mL streptomycin/penicillin at 37 ℃ and 5% CO_2_. At confluence, adherent cells were trypsinized, and recultured in fresh medium at a ratio of 1:2. Passage 5 was used in this experiment.

### Lentiviral vectors construction

*Homo* cDNAs of sTNFRII (GenBank: BT019927.1, 1-261 encoded amino acids) and Fc domain of immunoglobulin G1 (IgG1) (GenBank: AF027159.2) were synthesized by GeneChem (Shanghai, China). The sTNFRII-Fc fusion gene sequence was generated by combining the two segments with a flexible linker (5’-GGAGGTGGAGGATCA-3’). A total of 150 ng plasmid template was used to amplify and identify *sTNFRII-Fc* with PrimeSTAR® HS DNA polymerase (Takara, Japan). The amplification program was as follows: 95 ℃ for 5 min, followed by 30 cycles of 98 ℃ for 10 s, 55 ℃ for 10 s, 72 ℃ for 90 s and then 1 cycle of 72 ℃ for 8 min. Primers used for the amplification of *sTNFRII-Fc* were as follows: 5’-GAGGATCCCCGGGTACCGGTCGCCACCATGGCGCCCGTCGCCGTCTGG-3’ (forward) and 5’-TCCTTGTAGTCCATACCTTTACCCGGAGACAGGGAGAGGCTC-3’ (reverse). Amplified *sTNFRII-Fc* was then scanned with a Tanon-2500 gel imager system (Tanon, Shanghai, China).

The expression cassette of sTNFRII-Fc was cloned into the lentiviral vector GV358 (GeneChem, Shanghai, China) which contains the enhanced green fluorescent protein (EGFP) and puromycin resistance gene with restriction enzymes *Age*I (N-terminus) and *BamH*I (C-terminus), and the sequence was confirmed by DNA sequence analysis. The resulting lentiviral vector, LV-sTNFRII-Fc, was then cotransfected together with pHelper1.0 (containing *rev*, *pol* and *gag* gene of human immunodeficiency virus) and pHelper2.0 (containing a herpes simplex virus *VSV-G* gene) into 293 T cells using Lipofectamine 2000 Reagent (Thermo Fisher Scientific). Forty-eight hours after transfection, the virus-containing supernatants were collected, filtered (0.45-μm filters), and then concentrated by ultracentrifugation (25,000 rpm, 120 min, 4 ℃). After centrifugation, the pellet was resuspended by the virus preservation solution (GeneChem, Shanghai, China), and the transducing unit titer (TU/mL) of lentivirus was determined by quantitative polymerase chain reaction (qPCR) [28].

### Generation of sTNFRII-MSC stable cell line

Approximately 1 × 10^5^ UC-MSCs were seeded into six-well plate, and transfection was performed when UC-MSCs were reached 60% confluent. Culture medium was replaced with 1.5 mL of fresh medium containing lentivirus at a multiplicity of infection (MOI) of 7 pfu per cell with 10 μg/mL of polybrene (Sigma). The virus-containing medium was removed after 12 h transfection, and fresh DMEM/F12 medium containing 10% FBS was added. The cells were cultured at 37 ℃ with 5% CO_2_ for another 48 h, and then transferred to a 25-cm^2^ culture flask with 5 mL of fresh medium. Stable cell lines were generated by screening in 625 ng/mL of puromycin (Puro) (Sigma) for 2 weeks, and maintained in 300 ng/mL of puro, then used for experiments within the following 3 weeks. The infection efficiency was determined by flow cytometry and fluorescence microscope.

### Concentration of sTNFRII-Fc in supernatants

To detect the level of sTNFRII-Fc in supernatants, 2 × 10^5^ MSCs or transduced MSCs (including EGFP-MSC and sTNFRII-MSC) at passage 1 or 3 (after transfection) were cultured in six-well plates, and supernatants were collected at varying time-points. Concentrations of sTNFRII-Fc were determined by a human sTNFRII ELISA kit (R&D System, Minneapolis, MN) according to the instructions.

### Phenotype of sTNFRII-MSC

Surface antigens of sTNFRII-MSCs were analyzed by flow cytometry. Cells were trypsinized using 0.25% trypsin–EDTA (WISENT, Quebec, Canada), and incubated with 1% bovine serum albumin (BSA; Gibco) for 0.5 h to block non-specific antigen binding. Then, the cells were incubated with PE/APC-conjugated mouse anti-human CD90, CD73, CD105, CD45, CD34 and HLA-DR (Biolegend, USA) respectively for 1 h at 4 ℃. Then, cells were washed twice and suspended for flow cytometry.

### Tri-lineage mesenchymal differentiation assay

sTNFRII-MSCs were tested for multi-lineage differentiation potential using MSCgo™ differentiation kits (BI, Cromwell, CT, USA) for osteogenic, chondrogenic, and adipogenic differentiation according to the instructions. For evaluating the osteogenic ability of sTNFRII-MSCs, cells were fixed and stained with 2% alizarin red S (Sigma-Aldrich, MO, USA), and optical density (OD) of the eluent was measured at 550 nm after eluting with 10% cetylpyridinium chloride (Sigma) for 1 h at room temperature. Alcian blue staining for aggrecan (AGG) was used to assess the chondrogenic ability of sTNFRII-MSCs. Cells were fixed and stained with 1% alcian blue (Sigma) solution overnight at room temperature, and then de-stained with 8 M guanidine hydrochloride (Sigma) overnight at 4 ℃. The guanidine hydrochloride solutions were collected for the measurement of absorbance at 600 nm. For adipogenic differentiation, lipid vesicles were stained with oil red O for 30 min at room temperature, and then eluted using isopropanol for 1 h at room temperature. The absorbance of the isopropanol eluent was detected at 500 nm.

### Preparation of conditioned medium (CM)

Briefly, sTNFRII-MSC, EGFP-MSCs or MSCs at passage 5 were cultured with 5% CO_2_ and at 37 ℃ until 70–80% confluency. Then, fresh DMEM/F12 with less FBS (3%) was replaced to reduce the generation of toxins or uncalled proteins. The supernatant was collected after 48 h culture and filtrated using a 0.22-μm pore filter to remove the cell debris. The collected sTNFRII-MSC-CM, EGFP-MSC-CM or MSC-CM were stored at − 80 ℃ and used for subsequent coculture procedure.

### In vitro functionality of sTNFRII-MSC

#### Surface expression of intercellular adhesion molecule (ICAM)-1 and vascular cell adhesion molecule (VCAM)-1 on MSCs culturing with sTNFRII-MSC-CM

Glass coverslips were installed onto the bottom of 24-well plates before MSCs (5 × 10^3^ cells) were seeded, and cultured for another 12 h. sTNFRII-MSC-CM, EGFP-MSC-CM or MSC-CM plus with 20 ng/mL TNF-α (PeproTech, Rocky Hill, NJ, USA) were added into each well and incubated for 48 h. The cells were washed, fixed with 4% paraformaldehyde and blocked with 0.5% BSA. Then, ICAM-1 (Santa Cruz, 1:100) and VCAM-1 (Santa Cruz, 1:50) antibody were added and incubated overnight at 4 ℃. Anti-mouse Alexa Fluor 594 and anti-rabbit Alexa Fluor 488 were used as the secondary antibody. Glass coverslips from each well were taken out and fluorescent images were collected using a TCS SP8 confocal microscope (Leica, Wetzlar, Germany).

#### Expression of ICAM-1 and VCAM-1 on sTNFRII-MSC

A total of 2 × 10^5^ of sTNFRII-MSC, EGFP-MSCs or MSCs were stimulated with TNF-α (20 ng/mL) and/or IL-1β (PeproTech, 20 ng/mL) for 48 h. The expression of ICAM-1 and VCAM-1 on RA-FLS was determined by Western blotting. Briefly, cells were lysed in radioimmunoprecipitation assay (RIPA) buffer (Beyotime, China) for 30 min on ice and then centrifuged at 12,000 rpm for 30 min at 4 ℃. Supernatant of the lysates was mixed with SDS-PAGE (Beyotime, China) at the ratio of 5:1, then electrophoretically separated and transferred to a polyvinylidene fluoride (PVDF) membrane. The PVDF membranes were incubated with ICAM-1 (1:200) and VCAM-1 (1:100) antibody respectively overnight at 4 ℃. The dilution of corresponding secondary anti-rabbit/mouse antibody is 1:10,000. Membranes were then scanned with ImageQuant LAS 4000mini (GE Healthcare) and protein bands were quantified using ImageJ software (National Institutes of Health).

#### Receptor activator of nuclear factor κB ligand (RANKL) and osteoprotegerin (OPG) level in coculture system

A 0.4-μm transwell (Costar) was used to coculture RA-FLS and sTNFRII-MSC at the ratio of 2:1. RA-FLS (6 × 10^4^ cells) were seeded in the six-well plates and cultured overnight until adherence. Then, cells were washed with serum-free DMEM/F12, and 3 × 10^4^ of sTNFRII-MSC or EGFP-MSC suspensions were added into the upper chamber of the transwell. Meanwhile, TNF-α (20 ng/mL) or IL-1β (20 ng/mL) was added in the coculture system and incubated for 48 h. Culture supernatant was collected for detection of RANKL (Arigo Biolaboratories Corp) and OPG (R&D Systems) levels using commercial ELISA kits according to the manufacturer’s protocol.

### In vivo functionality of sTNFRII-MSC

As previously described, lipopolysaccharide (LPS)-induced cytokine storm was aroused in NOD/SCID mice [13, 29]. Briefly, LPS (Sigma) was dissolved in sterile water to a concentration of 3 mg/mL. One week before LPS challenge, mice were pretreated with sTNFRII-MSC (i.v., 5 × 10^6^ cells), EGFP-MSC (i.v., 5 × 10^6^ cells) and Etanercept (s.c., 4 mg/kg) respectively on Day 0 and Day 4 as shown in Fig. 2J. Then, mice received a sublethal dosage of LPS (20 mg/kg) via intraperitoneal injection on Day 7. Blood samples were harvested at different time points (0 h, 1.5 h and 6 h after LPS challenge), and centrifugated at 4 ℃, 1000 × g, 10 min. Serum was collected and stored at − 80 ℃ for quantification of sTNFRII, TNF-α, IL-1β and IL-6 levels using ELISA kits according to the instructions.

### Collagen-induced arthritis (CIA) model establishment, treatment and assessment

Collagen-induced arthritis was induced in 8-week-old male DBA/1 mice in conformity to experimental animal ethics committee of Anhui Medical University. Briefly, chick type II collagen (CII) (Chondrex, Redmond, WA, USA) was dissolved in 0.1 M glacial acetic acid overnight at 4 ℃. An equal volume of complete Freund’s adjuvant was mixed with CII solution to produce a final concentration of 2 mg/mL emulsion. To induce CIA, DBA/1 mice were injected intradermally at the back and the base of the tail with 100 μl of the prepared emulsion, followed by a booster immunization 21 days later. The day of the first immunization was defined as day 0. After the onset of arthritis (day 28), the mice received a single intravenous injection of 1 × 10^6^ sTNFRII-MSCs or non-engineered MSC in 150 μl saline by tail vein. Etanercept was served as positive control. Mice were administered 5 mg/kg of Etanercept (Guojian Pharmaceutical Co., Ltd., Shanghai, China) three times per week for 3 weeks after the booster immunization. The mice in normal and CIA group were given an equal volume of saline.

Body weight, arthritis index (AI) and swollen joint count (SCJ) were monitored every three days as reported [30]. For AI (16-point scale): 0 = normal; 1 = redness and/or swelling of the paw or one digit; 2, redness and/or swelling of two paws and joints; 3 = more than two joints involved; and 4 = All paws and digits involved with severe arthritis. Regarding SJC (24-point scale): every swollen phalanx joint or ankle joints is counted as 1 point, and thus, the maximum SJC score for each mouse was 24. Animals were sacrificed on day 49, blood and relevant organs were collected for further investigation.

### Histological evaluation of the ankle joints and spleens

The hind ankle joints and spleens of mice from each group were removed, washed and fixed in 4% paraformaldehyde at room temperature for 48 h. Regarding hind ankle joints, 10% EDTA (w/v) was used to decalcify the calcium from bone or cartilage. The samples were embedded in paraffin and sections (5-mm) were stain with H&E, Toluidine Blue (Sigma) and Alcian Blue & Alizarin red S (Sigma) respectively. Pathological changes in the spleen and ankles were evaluated by two blinded observers. Established scoring systems were as follows [30]: for ankle joints (5-point scale, 0 = no effect to 4 = severe effect), five parameters were involved, including inflammation, cartilage erosion, hyperplasia of synovium, mononuclear cells infiltration and formation of pannus; regarding spleens (4-point scale, 0 = no effect to 3 = severe effect), five parameters were involved, including the number of germinal centers (GCs), cellularity of the periarteriolar lymphoid sheath (PALS), lymphoid follicles, marginal zone, and red pulp hyperemia.

### Detection of thymus index and spleen index

The thymus and spleen were removed aseptically and placed in the centrifuge tubes. The weight of spleens and thymus were collected using a precision electronic scale and the thymus index or spleen index was shown as the ratios of thymus or spleen weight to mouse body weight (milligrams per gram).

### T and B cell proliferation assay

T cells were isolated from thymus whereas B cells were isolated from spleen of each mouse using lymphocyte separation medium (Dakewe Biotech). 1 × 10^6^ cells in 100 µl RPMI 1640 (5% FBS) were seeded into 96-well flat-bottom plates. Then, 3 mg/L concanavalin A (ConA, Sigma) and 4 mg/L lipopolysaccharide (LPS, Sigma) were respectively added to stimulate T and B lymphocytes proliferation. Cells were cultured for another 48 h at 37 ℃ with 5% CO_2_. one hour before the end of culture, A total of 10 µl Cell Counting Kit-8 (CCK-8) (Kumamoto) was added to each well. Then, the absorbance was read at 450 nm.

For collagen II-induced specific CD4^+^ T cell proliferation, CD4^+^ T cells of spleen of mice from each group were isolated using a cell sorter (BD FACSAria) [30]. Purified splenic CD4^+^ T cells (2 × 10^5^) in 100 µl RPMI 1640 (5% FBS) were seed into 96-well flat-bottom plates and then loaded with 20 µg/mL collagen II (Chondrex). After culture for 48 h, 10 µl of CCK-8 was added to each well and then incubated at 37 ℃ with 5% CO_2_ for two hours. Subsequently, the absorbance was read at 450 nm.

To evaluate the effects of apoptosis/autophagy on the immunosuppressive ability of MSCs, 2 × 10^5^ of CD4^+^ T cells isolated from spleen of CIA mice as described above were directly cocultured with MSCs or sTNFRII-MSC in 100 µl RPMI 1640 (5% FBS) at the ratio of 1:1. Before coculture, MSCs or sTNFRII-MSC were incubated with 20 ng/mL TNF-α + 10 µg/mL cycloheximide (CHX, Sigma) (sensitizes cells to TNF-α-induced apoptosis) for 12 h to induce apoptosis and autophagy, and 3-MA (5 mM, Sigma) were used as inhibitor of autophagy [31]. After coculture for 48 h, the suspended cells were transferred into a new 96-well plate with 100 µl serum-free RPMI 1640, and10 µl of CCK-8 was added to each well, then incubated at 37 ℃ with 5% CO_2_ for two hours. Subsequently, the absorbance was read at 450 nm.

### Analysis of T and B cell subsets in CIA mice

Splenic single cell suspension was harvested after incubation with FACS™ Lysing Solution (BD Pharmingen) for 10 min at room temperature. A total of 1 × 10^6^ cells in 250 µl PBS with 0.5% BSA were stained with fluorescence-conjugated anti-mouse monoclonal antibodies against CD4-FITC, CD25-APC, CD19-FITC, CD138-PE, CXCR5-APC and PD-1-PE (BD Pharmingen) at 4 ℃ for 30 min. For intracellular staining, the cells were stimulated with Leukocyte Activation Cocktail (BD Pharmingen) for 5 h and then fixed and permeabilized for 30 min using the Fixation/Permeabilization Kit (eBioscience). For foxp3 staining, cells were pretreated with Foxp3/Transcription Factor Staining Buffer Set (eBioscience) according to the instructions before intracellular staining. The permeabilized cells were incubated with fluorescence-conjugated anti-mouse IFN-γ-PE, IL-4-APC, IL-10-APC, IL-17-APC and Foxp3-PE (BD Pharmingen) for 30 min at 4 ℃ before detection. A flow cytometry (Cytoflex, Beckman Coulter) was used to analyze the percentage of CD4^+^IFN-γ^+^ T helper (Th) 1 cells, CD4^+^IL-4^+^ Th2 cells, CD4^+^IL-17^+^ Th17 cells, CD4^+^CD25^+^Foxp3^+^ regulatory T cells (Treg), CD4^+^CXCR5^+^PD-1^+^ follicular helper T cells (Tfh), CD19^−^CD138^+^ plasma cells and CD19^+^IL-10^+^ regulatory B cells (Breg).

### Analysis of cytokines and immunoglobulins in serum

The concentrations of cytokines (TNF-α, IFN-γ, IL-4, IL-10 and IL-17) (R&D Systems) and immunoglobulins (IgG1, anti-CII (Fcmacs Biotech) and IgG2a (RayBiotech)) were detected using commercial ELISA kits according to the manufacturers’ instructions.

### Immunohistochemistry

Ankle joints immunostaining was conducted to visualize the expression of collagen II, MMP-13, tissue inhibitors of metalloproteinase (TIMP)-1 and a disintegrin-like and metalloproteinase with thrombospondin motifs (ADAMTS)-5 on cartilage and synovium respectively. Sections were incubated with primary antibodies against collagen II (1:150 dilution, Abcam), MMP-13 (1:200 dilution, Novus), TIMP-1 (1:100 dilution, Abcam) or ADAMTS-5 (1:100 dilution, Novus) at 4 ℃ overnight and enzyme-labeled goat anti-mouse/rabbit IgG polymer (ZSGB-BIO, Beijing, China) was used as the secondary antibody. Sections were then visualized with 3,3’-diaminobenzidine. The percentages of positive cells or immunostaining intensity was used to assess the expression of relative proteins on joint cartilage and synovium. For cartilage, percentages of MMP-13, TIMP-1 and ADAMTS-5 positive chondrocytes were determined by counting the number of stained cells and dividing by the total number of chondrocytes, whereas collagen II expression was counted by immunostaining intensity. For synovium, immunostaining intensity was used to evaluate the abundance of MMP-13, TIMP-1 and ADAMTS-5.

### Immunofluorescence assay

CIA mice received 1 × 10^6^ of EGFP-MSC or sTNFRII-MSC transplantation via tail vein, and were sacrificed 1, 3, 7 and 14 days after injection respectively. The spleens and ankle joints were harvested and fixed in pre-cooled 4% paraformaldehyde for 12 h. After washing with PBS, samples were embedded using optimal cutting temperature compound (OCT). Sections (6-µm) were incubated with anti-human CD105 (1:100, Abcam) at 4 ℃ overnight. The second antibody was anti-human Alexa Fluor 594 for 1 h at 37 ℃. After DAPI staining, sections were observed with a fluorescence microscope (Leica, Wetzlar, Germany).

For detection of apoptosis and autophagy of sTNFRII-MSC in vivo, spleen sections (d14) were incubated with anti-human CD105 (1:100) and cleaved caspase 3 (1:150, Abcam)/LC3B-II (1:200, Abcam). The second antibody was anti-human Alexa Fluor 594 for 1 h at 37 ℃. After DAPI staining, sections were observed with a TCS SP8 confocal microscope (Leica, Wetzlar, Germany).

### Apoptosis detection

MSCs apoptosis was induced by 20 ng/mL TNF-α and 10 µg/mL CHX (sensitizes cells to TNF-α-induced apoptosis) at different time points (6 h, 12 h and 24 h) [31]. For comparison of anti-apoptosis effect of MSC, EGFP-MSC and sTNFRII-MSC, MSCs were divided into three groups: control group (cultured with MSC-CM), EGFP-MSC group (cultured with EGFP-MSC-CM) and sTNFRII-MSC group (cultured with sTNFRII-MSC-CM). Cells were treated with 20 ng/mL TNF-α and 10 µg/mL CHX for 12 h. At the end of the culture, cells were collected and stained with the combination of annexin V-FITC and propidium iodide (PI)-PE (Annexin V-FITC/PI Apoptosis Detection Kit, BestBio, China) for 30 min. Apoptosis rates were detected by flow cytometry (Cytoflex, Beckman Coulter) and representative fluorescent images were collected by an imaging flow cytometer (ImageStream^X^ Mark II).

### Western blot analyses

MSCs were treated with 20 ng/mL TNF-α and 10 µg/mL CHX at different time points (6 h, 12 h and 24 h). For comparison of anti-apoptosis effect of MSC, EGFP-MSC and sTNFRII-MSC, cells were treated with 20 ng/mL TNF-α and 10 µg/mL CHX for 12 h. Cell total protein was harvested after lysing and centrifuging at 12,000 × g for 15 min at 4 ℃. Supernatant was collected and added with 5 × protein loading buffer (Beyotime Biotechnology, China), then the protein samples were denatured at 100 ℃ for 10 min. The denatured protein samples were separated by polyacrylamide gel electrophoresis and transferred electrophoretic ally to a polyvinylidene fluoride membrane (Millipore, MA, USA). The dilution of primary antibody of Bcl-2, Bax, Caspase 3, Cleaved Caspase 3, Caspase 8, Cleaved Caspase 8, LC3B I/II, TRIB3 and β-actin (all from Proteintech, USA) is 1:800. The dilution of second antibody of goat anti-rabbit/mouse is 1:10,000 (ZSGB-BIO, Beijing, China). The membranes were scanned with an ImageQuant LAS 4000mini (GE Healthcare) and the density of protein bands was analyzed by ImageJ software (National Institutes of Health).

### Immunogenicity detection

2 × 10^5^ of MSCs were cultured with sTNFRII-MSC-CM, EGFP-MSC-CM and MSC-CM respectively in the presence of 20 ng/mL TNF-α + 20 ng/mL IFN-γ. After culturing for 48 h at 37 ℃ and 5% CO_2_, cells were collected and stained with fluorescence-conjugated anti-human monoclonal antibodies against HLA-DR for 30 min at 4 ℃. Then, cells were washed twice and analyzed by flow cytometry (Cytoflex, Beckman Coulter).

### Chondrogenic differentiation detection

MSC**,** EGFP-MSC and sTNFRII-MSC were cultured in chondrogenic medium with or without TNF-α (20 ng/mL) for 14 days. Chondrogenic ability was assessed by alcian blue staining and western blot as described above. The dilution of primary antibody of aggrecan (AGG) (Novus) and SOX-9 (Novus) was 1:500.

### Statistical analysis

Data in figures are shown as the means ± SD. ANOVA (SPSS Software Products) was used to analyze the data from multiple groups. A p values < 0.05 with a 95% confidence interval were considered significant.

## Results

### sTNFRII-Fc-expressing MSCs: lentiviral vector construction, transfection and expression

**Fig. 1:**
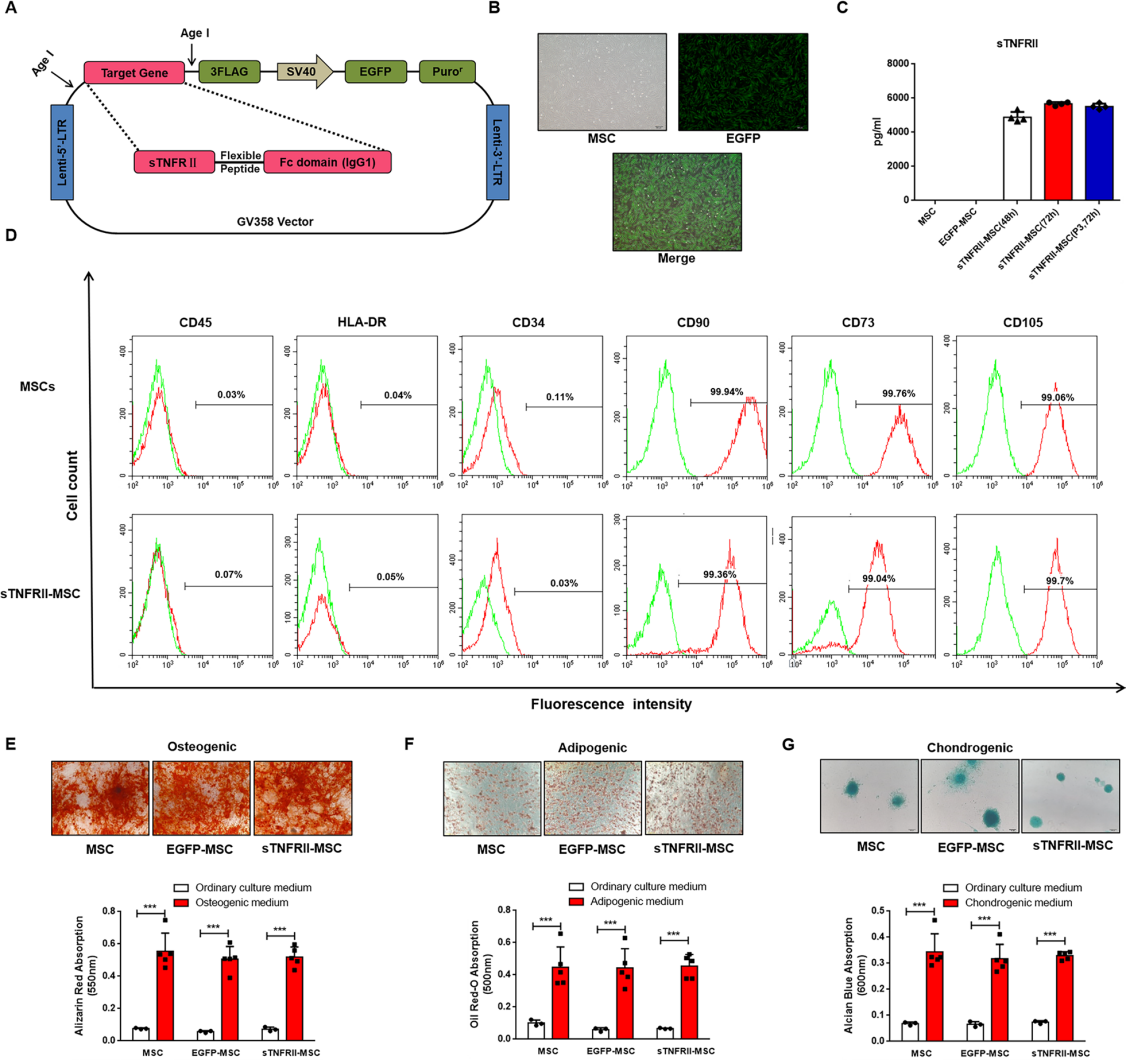
Construction of sTNFRII-MSC. **a** Schematic representation of the GV358 lentivirus vector. Transcription of the sTNFRII-Fc, EGFP and Puro^r^ gene is initiated from the 5’ LTR. The vector is not drawn to scale. **b** The morphology and transfection efficiency of UC-MSCs. UC-MSCs were infected with LV-sTNFRII-Fc, and the expression of EGFP were monitored under a fluorescence microscope. Representative pictures were shown 48 h after transfection. Scale bars: 500 μm. **c** Concentration of sTNFRII-Fc in the supernatant of sTNFRII-MSC. Supernatant of UC-MSC, EGFP-MSC and sTNFRII-MSC were collected after 48/72 h culture, and level of sTNFRII-Fc was determined using ELISA (n = 4). **d** Immunophenotypic profile of sTNFRII-MSC. Flow cytometry results showed that sTNFRII-MSC (red lines) were negative for CD45, HLA-DR and CD34, and positive for CD90, CD73 and CD105. Negative controls are represented by the green lines (n = 5). **e** Osteogenic capacity of sTNFRII-MSC. Alizarin Red S staining was used to evaluate the osteogenic capacity of sTNFRII-MSC, and the absorbance of eluent was determined at 550 nm for semiquantitation (n = 3–5). **f** Adipogenic capacity of sTNFRII-MSC. Oil Red staining was used to evaluate the adipogenic capacity of sTNFRII-MSC, and the absorbance of eluent was determined at 500 nm for semiquantitation (n = 3–5). **g** Chondrogenic capacity of sTNFRII-MSC. Alcian Blue staining was used to evaluate the chondrogenic capacity of sTNFRII-MSC, and the absorbance of eluent was determined at 600 nm for semiquantitation (n = 3–5). Data are presented as the mean ± SD values. ***P < 0.001

*sTNFRII-Fc* was generated by combining *sTNFRII* and *IgG1-Fc*, and sequence was confirmed by DNA sequence analysis (Additional fi1e 1: Fig. 1A-C). The expression cassette of sTNFRII-Fc was cloned into the lentiviral vector GV358 containing EGFP and puromycin resistance gene (Fig. 1a), and gel electrophoresis revealed that a characteristic band was shown at 1.54 kb (Additional fi1e 1: Fig. 1D) which consistent with our predictions.

**Fig. 2:**
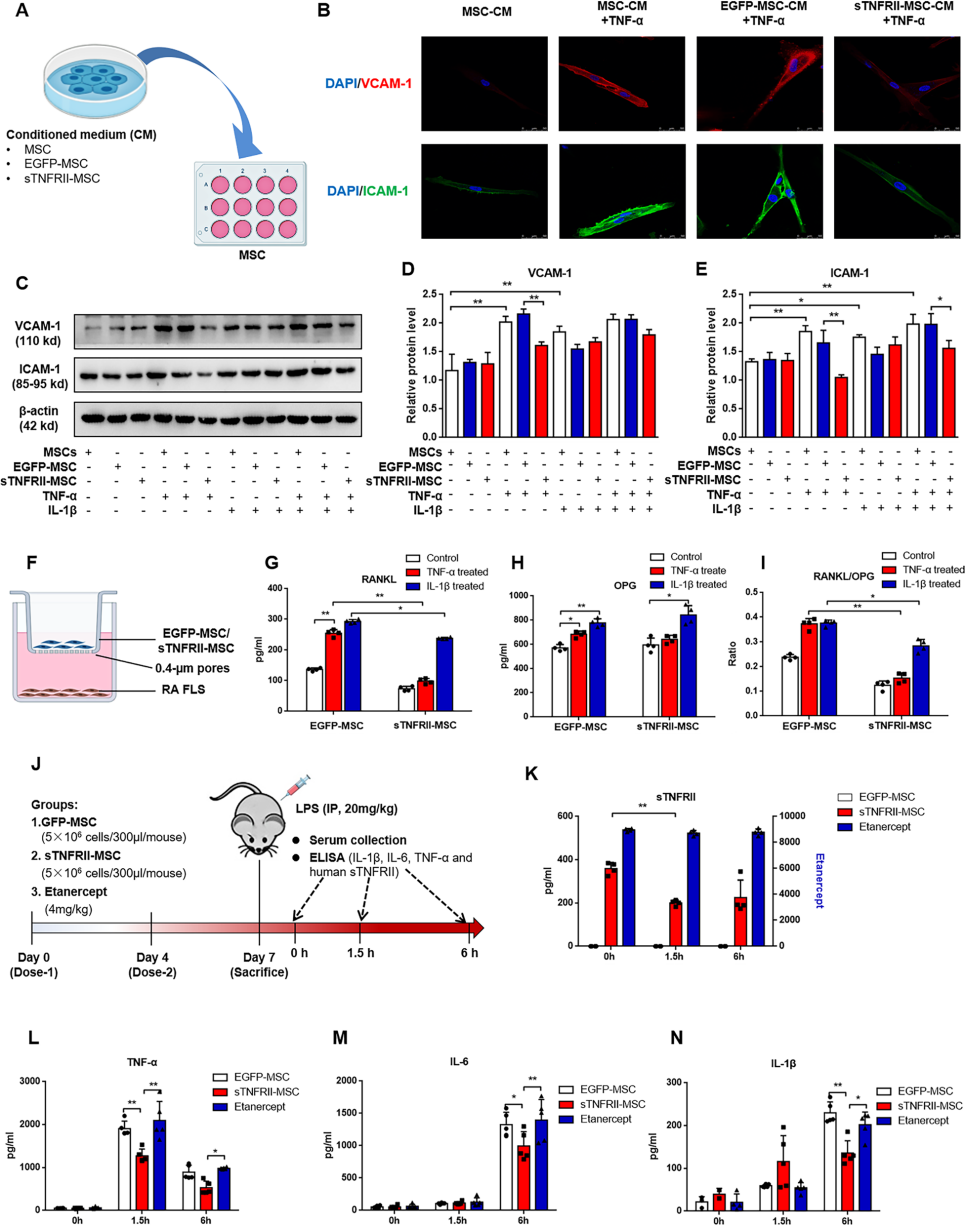
In vitro and vivo functionalities of sTNFRII-MSC. **a** Schematic representation of in vitro incubation of MSCs with conditioned medium (CM) from MSCs, EGFP-MSC or sTNFRII-MSC. **b** Immunofluorescence of MSCs incubated with CM from MSCs, EGFP-MSC or sTNFRII-MSC with/without TNF-α. Cells were then stained for VCAM-1 (red), ICAM-1 (green) and DAPI (blue) (n = 3). Scale bars: 10 µm. **c** Western blot analysis showing protein levels of VCAM-1, ICAM-1 in sTNFRII-MSC stimulated with/without TNF-α and/or IL-1β. **d**, **e** Densitometry quantification of western blot from (**c**). The intensity of each was normalized to β-actin intensity (n = 3). **f** Schematic representation of the coculture study. RA FLS (lower chamber) was cocultured with sTNFRII-MSC (upper chamber) using transwell coculture system and the supernatant was collected for RANKL and OPG detection. **g**–**i** Experiments were performed as **f**. Levels of RANKL (**g**) and OPG (**h**) were detected using ELISA, and RANKL/OPG ratio (**i**) was analyzed (n = 4). **j** Schematic representation for validating the in vivo functionality of sTNFRII-MSC. **k**–**n** Experiments were performed as **j**. Effect of sTNFRII-MSC intervention on the concentration of sTNFRII-Fc (**k**), TNF-α (**l**), IL-6 (**m**) and IL-1β in serum of NOD/SCID mice after LPS challenge (n = 4–5). Data are presented as the mean ± SD values. *P < 0.05, **P < 0.01

Constructed sTNFRII-Fc lentivirus was used to infect human UC-MSCs and sTNFRII-MSC stable cell line was generated via purification with puromycin-containing medium. Figure 1c shows that infected UC-MSCs expressed green fluorescence, and almost all the cells were transfected successfully. To further determine the transfection efficiency, flow cytometry was used to quantify the proportion of EGFP positive cells, and the transfection efficiency was above 98% after puromycin purification (Additional fi1e 1: Fig. 2). We next examined whether the transfected sTNFRII-MSC could synthesize and secret the fusion protein. sTNFRII-Fc was strongly detected in the culture supernatant of sTNFRII-MSC, but not in untransfected cells or cells transfected with empty vectors. Moreover, sTNFRII-MSC from the third generation could also express high level of sTNFRII-Fc (Fig. 1c).

### sTNFRII-MSC retain the inherent biological characteristics of MSCs

To investigate whether sTNFRII-Fc transfection mediated by lentivirus would alter the inherent biological characteristics of MSCs, immunophenotyping and tri-lineage mesenchymal differentiation assay were conducted. Expression of CD90, CD73 and CD105 (positive marker for MSCs) were highly detected in sTNFRII-MSC, and almost no CD45, CD34 and HLA-DR (negative marker for MSCs) could be detected in sTNFRII-MSCs, which is in line with the results of MSCs (Fig. 1d). In addition, the potential of osteoblast, chondrocytes and adipocytes differentiation ability was determined in MSCs, EGFP-MSC and sTNFRII-MSC. After a 14-day-induction in osteogenic medium, the extracellular calcium deposits were semi-quantified after alizarin red S staining which was similar among the three cell batches (Fig. 1e). For adipogenic differentiation, oil-red O staining was conducted to discern the lipid droplets after a 20-day-induction in adipogenic medium, and the OD of the eluent from sTNFRII-MSC was similar to that observed for MSCs and EGFP-MSC (Fig. 1f). Moreover, the OD of eluent after alcian blue staining for AGG after chondrogenic induction were comparable among MSCs, EGFP-MSC and sTNFRII-MSC (Fig. 1g). These results suggested that MSCs retain the inherent immunophenotyping and tri-lineage mesenchymal differentiation capacity after sTNFRII-Fc transfection mediated by lentivirus.

### In vitro functionality of sTNFRII-MSC

To determine whether the sTNFRII-Fc secreted by sTNFRII-MSC has biological activity, CM harvested from MSCs, EGFP-MSC or sTNFRII-MSC was used to culture MSCs with/without TNF-α (Fig. 2a). It has been demonstrated that the treatment of MSCs with either TNF-α or IL-1β induces upregulated cellular surface expression of ICAM-1 and VCAM-1 [32, 33]. MSCs were stimulated with TNF-α for 24 h in the presence of CM harvested from MSCs, EGFP-MSC or sTNFRII-MSC, and then ICAM-1 and VCAM-1 expression was measured by laser confocal microscope. TNF-α significantly upregulated the expression of ICAM-1 and VCAM-1 of MSCs, whereas ICAM-1 and VCAM-1 expression from sTNFRII-MSC-CM treatment group, but not from those with MSC-CM or EGFP-MSC-CM treatment, was almost completely inhibited in the presence of TNF-α (Fig. 2b). Furthermore, we use TNF-α and/ IL-1β to directly stimulate MSCs, EGFP-MSC and sTNFRII-MSC, 48 h later, expression of ICAM-1 and VCAM-1 were detected by western blot. Both TNF-α and IL-1β or their combination could significantly upregulate the expression of ICAM-1 and VCAM-1 in MSCs and EGFP-MSC. sTNFRII-MSC could resist the upregulation of ICAM-1 and VCAM-1 induced by TNF-α, but not induced by IL-1β. In addition, sTNFRII-MSC partly resist the upregulation of ICAM-1 induced by combination of TNF-α and IL-1β (Fig. 2c–e).

Previous published results have demonstrated that TNF-α or IL-1β is responsible for the upregulation of RANKL in FLS [34, 35]. We used this fact to determine whether exposure to TNF-α or IL-1β would result in RANKL expression in RA-FLS when cocultured with sTNFRII-MSC. Additionally, we detected OPG (RANKL’s decoy receptor) levels and analyzed RANKL/OPG ratio in the coculture system (Fig. 2f). sTNFRII-MSC inhibited RANKL levels in the supernatant induced by TNF-α, but not induced by IL-1β (Fig. 2g). Although, both TNF-α and IL-1β could increase the RANKL and OPG levels (Fig. 2h) in the supernatant from coculture system, interestingly, sTNFRII-MSC downregulated RANKL/OPG ratio compared with EGFP-MSC (Fig. 2i). The above results support the conclusion that the sTNFRII-Fc fusion protein secret by sTNFRII-MSC is biologically active and capable of binding and neutralizing TNF-α, and thus block its biological functions.

### In vivo functionality of sTNFRII-MSC

Elevated TNF-α levels have been reported as a vanguard in various models of acute inflammation followed by increasing in other pro-inflammatory cytokines (e.g., IL-6 and IL-1β). Here, intraperitoneal injection of sublethal dose of LPS was conducted to induce cytokines storm in NOD/SCID mice to evaluate the in vivo functionality of sTNFRII-MSC. One week before LPS challenge, mice were received transplantation of 5 × 10^6^ sTNFRII-MSC or EGFP-MSC on day 0 and day 4, and mice treated with Etanercept were served as control. Mice were sacrificed at 0, 1.5 and 6 h post-LPS challenge, and serum was collected for detecting human sTNFRII, mouse TNF-α, mouse IL-1β and mouse IL-6. Experimental outline is shown in Fig. 2j. The results showed that mouse TNF-α in serum increased dramatically, peaking at 1.5 h after LPS injection, and an obvious reduction was observed in mice pretreated with sTNFRII-MSC compared with EGFP-MSC and Etanercept treatment. At 6 h after LPS injection, the level of mouse TNF-α began to decrease, and the concentration of mouse TNF-α in sTNFRII-MSC-pretreated mice was significantly lower than that in Etanercept-pretreated mice (Fig. 2l). At 1.5 h post-LPS challenge, there was no significant change in the levels of mouse IL-1β and IL-6 among the groups, but rose at 6 h after LPS injection. Moreover, significantly less mouse IL-1β and IL-6 were noted in mice transplanted with sTNFRII-MSC at 6 h post-LPS challenge (Fig. 2m, n). The concentration of human sTNFRII decreased at 1.5 h post-LPS challenge, companying with reduction in mouses TNF-α in animals receiving sTNFRII-MSC, whereas in Etanercept pretreatment group, human sTNFRII maintained at a high level (8000–10,000 pg/mL) (Fig. 2k). No human sTNFRII was detected in EGFP-MSC group. These results indicated that the concomitant decrease of mouse TNF-α and human sTNFRII is a result of bonding of these two molecules and subsequent clearance of the TNF-α-sTNFRII-Fc immunocomplex. Furthermore, the decreased TNF-α in circulation is responsible for the reduction of other pro-inflammatory cytokines (IL-1β and IL-6). For the reason why sTNFRII-MSC exhibited stronger capacity in inhibiting TNF-α than Etanercept, it appears that the sTNFRII-MSC not only neutralizes TNF-α by secreting sTNFRII-Fc, but acts by modulating nature killer cells and neutrophils (NOD/SCID mice are born with defects in T and B cells) that produce TNF-α [36].

### Transplantation of sTNFRII-MSC attenuates clinical signs of CIA mice

**Fig. 3:**
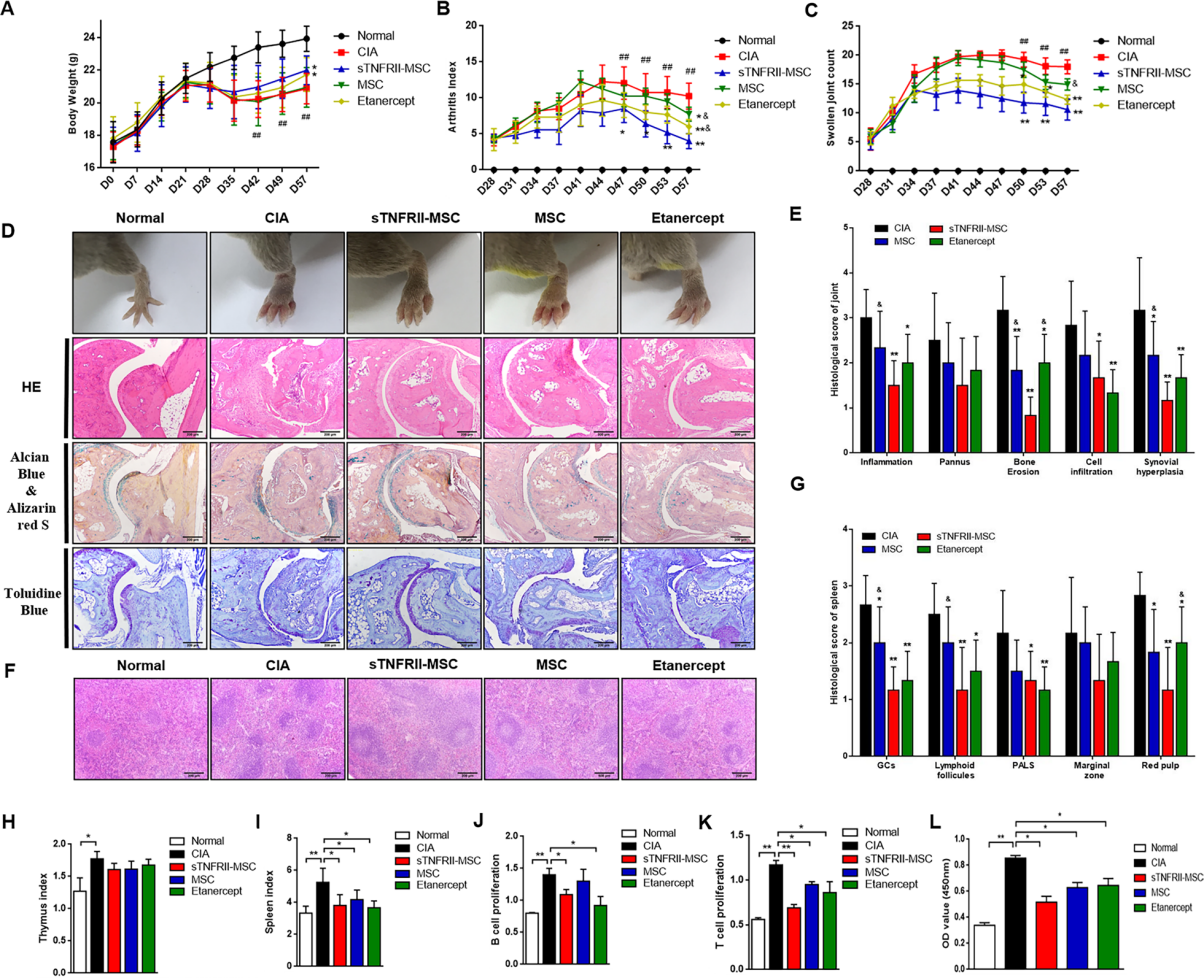
Transplantation of sTNFRII-MSC alleviates the pathological signs of CIA in mice. sTNFRII-MSC was transplanted into CIA mice after the onset of arthritis. **a** Body weight was measured every week from day 0 to 57 (n = 8). **b**, **c** The AI (**b**) and SJC (**c**) were monitored every 3 d from day 28 to 57 (n = 8). ^##^*p* < 0.01 versus normal group; *p < 0.05, **p < 0.01 versus CIA group; ^&^*p* < 0.05 versus sTNFRII-MSC group; **d** Representative photomicrographs of joint general appearance and histopathology. Scale bars: 100 μm. **e** Evaluation of histopathologic grading of joints (n = 5). *p < 0.05, **p < 0.01 versus CIA group; ^&^*p* < 0.05 versus sTNFRII-MSC group. **f** Representative photomicrographs of spleen histopathology. Scale bars: 100 μm **g** Evaluation of histopathologic grading of spleen (n = 5). *p < 0.05, **p < 0.01 versus CIA group; ^&^*p* < 0.05 versus sTNFRII-MSC group. **h**, **i** The effect of sTNFRII-MSC on thymus index (**h**) and spleen index (**i**) was shown based on the ratios of thymus/spleen weight to mouse body weight (n = 8). **j**, **k** The effect of sTNFRII-MSC on B cell (**j**) and T cell (**k**) proliferation induced by Con A and LPS separately in CIA mice was detected by CCK-8 assays (n = 8). **l** The effect of sTNFRII-MSC on specific chick type II collagen induced splenic CD4^+^ T cell proliferation (n = 5). Data are presented as the mean ± SD values. *p < 0.05, **p < 0.01

Well-established mice CIA model was used to evaluate the effects of sTNFRII-MSC on joint inflammation. After the onset of arthritis on day 28, mice were received i.v. injection of sTNFRII-MSC or MSCs, then body weight, AI and SJC were monitored every three days. As shown in Fig. 3a, the CIA mice gained weight more slowly than normal mice. However, MSCs failed to result in a recovery of body weight. In contrast, treatment with sTNFRII-MSC or Etanercept significantly restored the body weight on day 57 compared with that in the CIA group. For evaluating the development of arthritis, AI and SJC were measured from day 28 to day 57. The results showed that sTNFRII-MSC, MSCs and Etanercept significantly decreased the AI and SJC of CIA mice (Fig. 3b, c). In addition, sTNFRII-MSC exhibited stronger effect on decreasing AI of CIA mice than MSCs and Etanercept on day 57, and were more powerful in reducing SJC than MSCs. These results thus indicated that sTNFRII-MSC inhibited the progression of arthritis in mice and possessed potent anti-arthritic activity.

### sTNFRII-MSC improves articular and splenic histopathology in CIA mice

Swollen Joints were observed in all CIA mice, and sTNFRII-MSC, MSCs and Etanercept relieved joints swelling in different degrees. Results of pathologic examinations of ankle joints showed significant differences between sTNFRII-MSC and CIA group. More severe pathological manifestations, with inflammatory cell infiltration, cartilage destruction, and synovium hyperplasia, was observed in CIA ankle joints. sTNFRII-MSC administration strongly inhibited synovium hyperplasia, whereas MSCs and Etanercept had mild effects. Cartilage specific staining (Alcian Blue & Alizarin red S and Toluidine Blue staining) revealed stronger protective effect of sTNFRII-MSC on cartilage erosion and joint destruction than MSCs and Etanercept, and almost no bone erosion was observed in the group that received sTNFRII-MSC (Fig. 3d, e).

H&E staining of the spleens showed significant red pulp hyperemia, lymphoid follicles hyperplasia and GCs formation affected the spleens of CIA mice. In contrast, only minimal pathologic changes were observed in sTNFRII-MSC-treated mice. In comparison, sTNFRII-MSC was more powerful in improving lymphoid follicles hyperplasia and GCs formation than MSCs, and red pulp hyperemia than Etanercept (Fig. 3f, g).

### sTNFRII-MSC inhibits spleen index and splenic/thymic lymphocytes proliferation in CIA mice

The spleen and thymus indexes were notably elevated in CIA mice, and sTNFRII-MSC transfer reduced spleen index (Fig. 3i), whereas had no significant effect on thymus index in CIA mice (Fig. 3h). The proliferation of thymic T cells and splenic B cells was detected by CCK-8 assays. As illustrated in Fig. 3j and k, increased LPS-induced thymic T cell and ConA-induced splenic B cell proliferation were observed in CIA mice compared with normal mice. Implantation of sTNFRII-MSC caused an obvious reduction in T- (Fig. 3k) and B cell (Fig. 3j) proliferation. Collagen II-specific CD4^+^ T cells are essential for CIA pathology, we asked whether the ameliorating effects of sTNFRII-MSC on CIA mice were associated with the inhibition of pathogenic CD4^+^ T cell activation. CD4^+^ T cells were sorted from spleens of mice and stimulated with Collagen II. The result showed that sTNFRII-MSC notably inhibited the collagen II-specific CD4^+^ Tcell proliferation (Fig. 3l). Above data indicated that the effects of sTNFRII-MSC might be closely related to T cell-mediated cellular immunity and B cell-mediated humoral immunity.

### sTNFRII-MSC modulates T- and B cell subsets in CIA mice

**Fig. 4:**
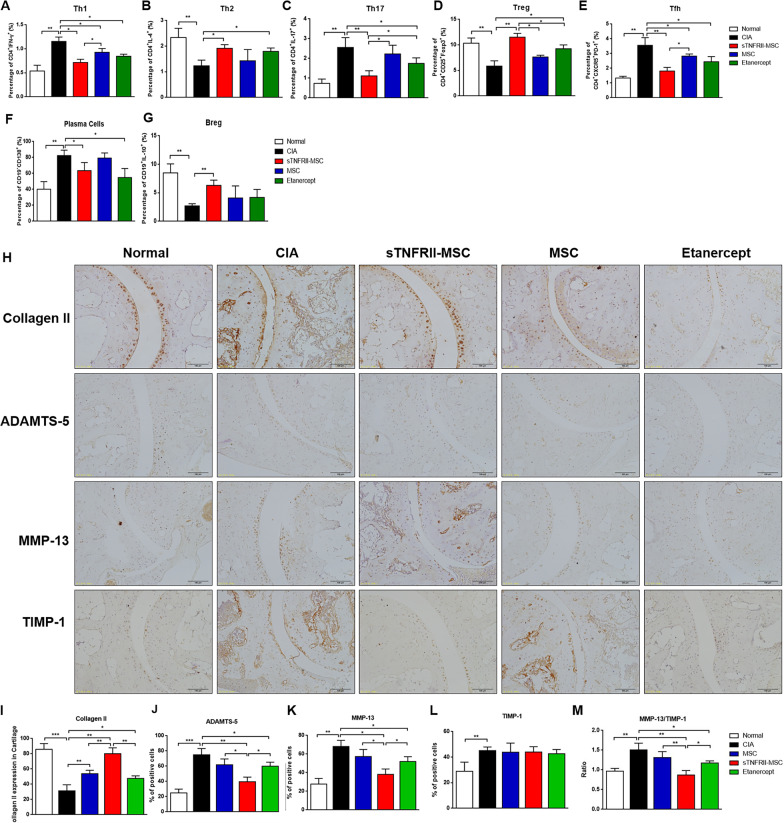
Transplantation of sTNFRII-MSC regulates the T and B cell subsets balance and ankle cartilage homeostasis in CIA mice. **a**–**e** sTNFRII-MSC regulates T cell subsets balance in CIA mice. sTNFRII-MSC transplantation inhibited the percentage of pathogenic Th1 (**a**), Th17 (**c**) and Tfh (**e**) cells but increased the percentage of regulatory Th2 (**b**) and Tregs (**d**) in the spleens of CIA mice (n = 8). **f**, **g** sTNFRII-MSC regulates B cell subsets balance in CIA mice (n = 8). sTNFRII-MSC transplantation inhibited the percentage of pathogenic plasma cells (**f**) but increased the percentage of regulatory Breg (**g**) in the spleens of CIA mice. **h** Immunohistochemical staining of collagen II, ADAMTS-5, MMP-13 and TIMP-1 in cartilage of ankle joints from normal, CIA mice and mice treated with sTNFRII-MSC. Scale bars: 100 μm. **i**–**l** Immunopositive cells were counted individually in three different areas (n = 4). The number of positively stained cells for collagen II (**i**), ADAMTS-5 (**j**), MMP-13 (**k**) and TIMP-1 (**l**) was averaged. The proportion of immunopositive cells was determined by dividing the number of positive cells by the total number of cells within the cartilage field. **m** The ratio of MMP-13/TIMP-1 in cartilage of ankle joints from CIA mice was analyzed. Data are presented as the mean ± SD values. *p < 0.05, **p < 0.01, ***p < 0.001

In RA, imbalances in T and B cell subsets differentiation, resulting in populations that are skewed toward pathogenic Th1, Th17, Tfh and plasma cells population rather than regulatory Th2, Treg and Breg, thus we next investigated whether sTNFRII-MSC directed T and B cell subsets (Additional fi1e 1: Fig. 3A-G). We observed that the frequency of CD4^+^IFN-γ^+^ Th1, CD4^+^IL-17^+^ Th17, CD4^+^CXCR5^+^PD-1^+^ Tfh and CD19^−^CD138^+^ plasma cells in the spleen of CIA mice were far more than that of normal mice, whereas CD4^+^IL-4^+^ Th2, CD4^+^CD25^+^Foxp3^+^ Treg and CD19^+^IL-10^+^ Breg populations were decreased in CIA mice. Transplantation of sTNFRII-MSC significantly reduced the percentage of Th1 (Fig. 4a), Th17 (Fig. 4c), Tfh (Fig. 4e) and plasma cells (Fig. 4f) in CIA mice, while restored Th2 (Fig. 4b), Treg (Fig. 4d) and Breg (Fig. 4g) to almost normal levels. Additionally, a stronger inhibitory effect on Th1, Th17, Tfh subsets was observed for sTNFRII-MSC compared with that with MSCs. Furthermore, sTNFRII-MSC implantation was more effective in restoring Treg subset than MSCs and Etanercept, and Th2 subsets than MSCs. In contrast, MSCs failed to reduce the percentage of plasma cells, and have no effect on restoring Th2 and Breg populations. These results thus suggested that sTNFRII-MSC’s therapeutic effects on mice CIA were closely related to rectifying the imbalance of T and B cell subsets, further sTNFRII-MSC is more effective in modulating immune homeostasis than MSCs and Etanercept in some respects.

### sTNFRII-MSC protects against cartilage matrix degradation in CIA mice

Cartilage destruction is a typical pathological manifestation of RA, which was induced both by FLS and chondrocytes, thus we next assessed the cartilage protective capacity of sTNFRII-MSC. Immunohistochemical analysis showed decreased collagen II, but increased ADAMTS-5, MMP-13 and TIMP-1 expression were observed in cartilage from CIA mice (Fig. 4h). sTNFRII-MSC notably restored collagen II (Fig. 4i), whereas downregulated the expression of ADAMTS-5 (Fig. 4j) and MMP-13 (Fig. 4k) in CIA cartilage. In comparison, the expression of collagen II was higher, but ADAMTS-5 and MMP-13 expression was lower in sTNFRII-MSC treated mice than MSCs or Etanercept treated mice. Additionally, the sTNFRII-MSC showed stronger inhibitory effect on abnormally elevated MMP-13/TIMP-1 ratio in cartilage from CIA mice compared with MSCs and Etanercept (Fig. 4m). Similar trend was observed in synovium tissues, which sTNFRII-MSC more remarkably reduced ADAMTS-5, MMP-13 and MMP-13/TIMP-1 ratio than MSCs (Additional fi1e 1: Fig. 4A-E). Taken together, these results demonstrated that sTNFRII-MSC protects cartilage by restoring the synthesis-metabolism balance of cartilage matrix.

### sTNFRII-MSC regulates serum cytokines and autoantibodies production in CIA mice

**Fig. 5:**
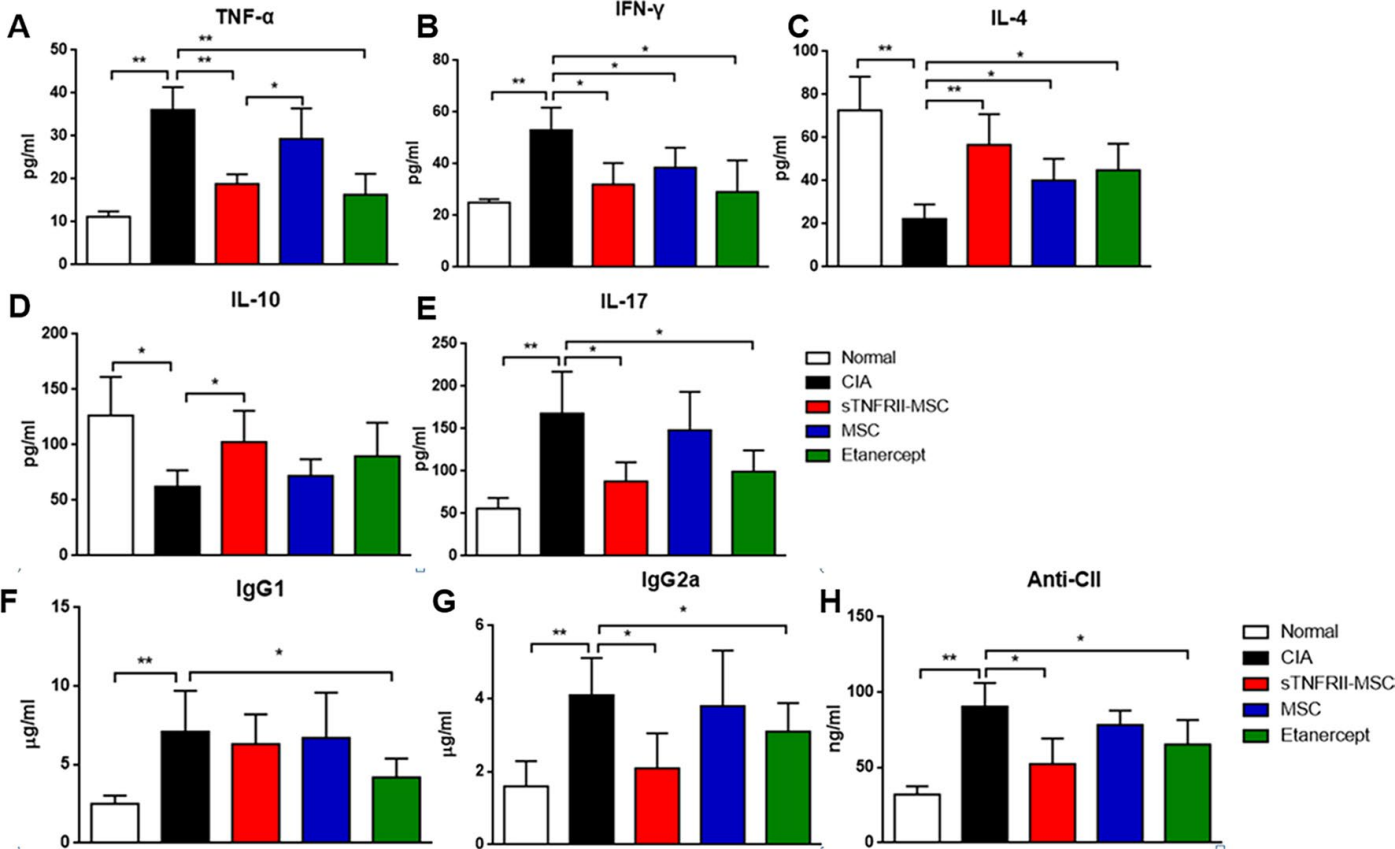
Transplantation of sTNFRII-MSC regulates the cytokine and autoantibody production in CIA mice. **a**–**e** Cytokine levels in serum of CIA mice transplanted with sTNFRII-MSC. TNF-α (**a**), IFN-γ (**b**), IL-4 (**c**), IL-10 (**d**), and IL-17 (**e**) levels in the serum of CIA mice were determined by ELISA (n = 5). **f**–**h** Autoantibody levels in serum of CIA mice transplanted with sTNFRII-MSC. Levels of IgG1 (**f**), IgG2a (**g**) and anti-CII autoantibody (**h**) in the serum of CIA mice were determined by ELISA (n = 5). Data are presented as the mean ± SD values. *p < 0.05, **p < 0.01

It has been well-documented that the imbalance between pro-inflammatory and anti-inflammatory cytokines are favorable to induce autoimmunity in RA. To explore the inflammation-regulating effect of sTNFRII-MSC, we measured the concentration of pro-inflammatory cytokines (TNF-α, IFN-γ and IL-17) and anti-inflammatory cytokines (IL-4 and IL-10) in the sera of mice. In CIA mice, levels of TNF-α, IFN-γ and IL-17 were upregulated. sTNFRII-MSC or Etanercept markedly reduced the level of TNF-α (Fig. 5a), IFN-γ (Fig. 5b) and IL-17 (Fig. 5e) in CIA mice, whereas MSCs showed no significant effect in decreasing TNF-α and IL-17. In contrast, concentration of IL-4 and IL-10 were decreased in CIA mice and either sTNFRII-MSC, MSCs or Etanercept increased the production of IL-4 (Fig. 5c), whereas an obvious increasing in IL-10 was only observed in the sTNFRII-MSC treated mice (Fig. 5d).

Autoantibodies are predominantly secreted by plasma cells, and considering that sTNFRII-MSC reduced the percentage of plasma cells in CIA mice, we further investigated the level of IgG1, IgG2a and anti-collagen II (CII) in the sera of mice. Although implantation of sTNFRII-MSC showed no obvious effect on downregulating IgG1 (Fig. 5f), they remarkably reduced the CD4^+^ T- cell-specific IgG2a and cartilage-specific anti-CII levels in CIA mice (Fig. 5g, h). However, MSCs administration failed to reduce either IgG1, IgG2a or anti-CII levels in sera of CIA mice.

### Tracking of the intravenously-injected sTNFRII-MSC

**Fig. 6:**
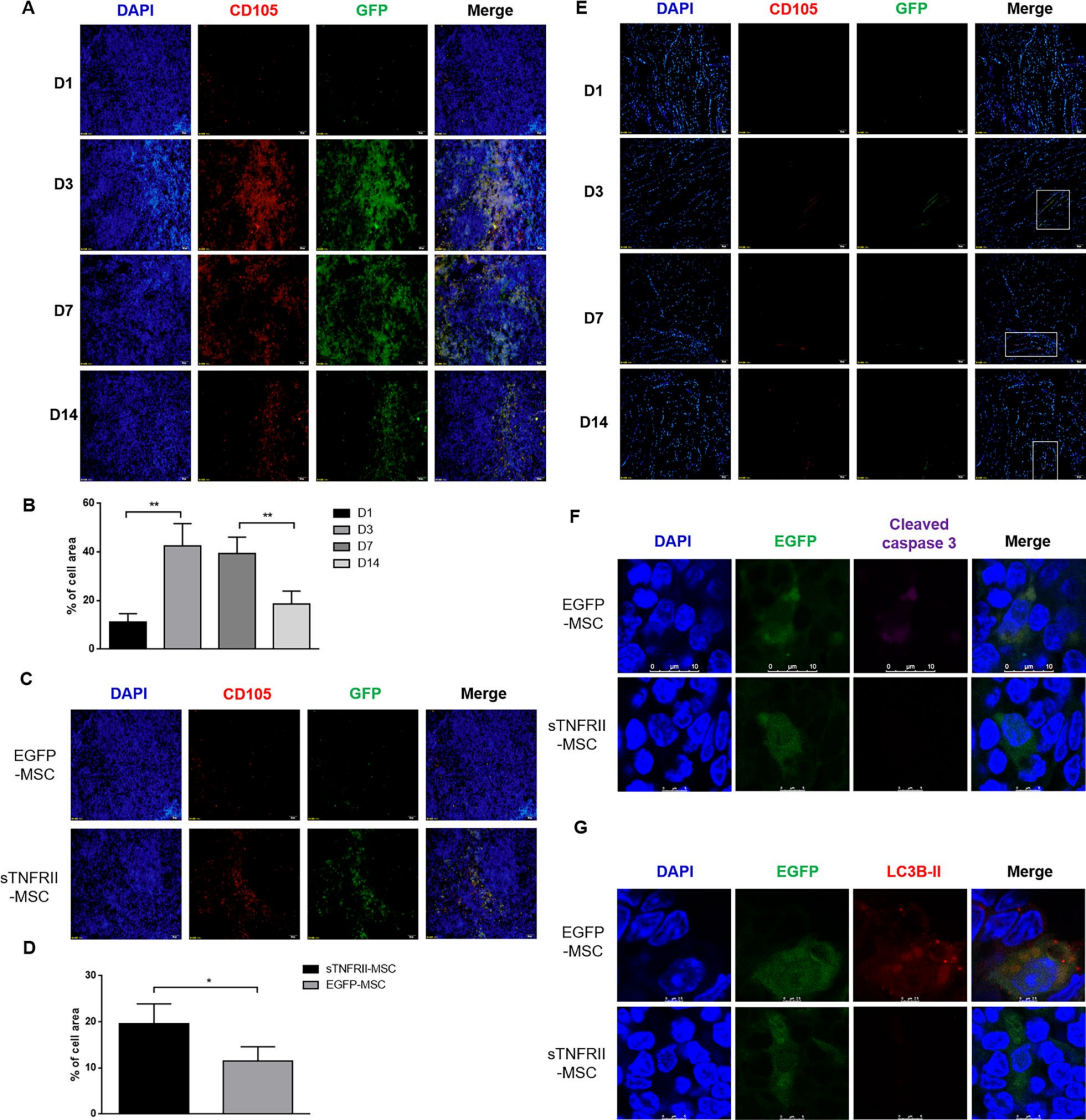
Detection of sTNFRII-MSC in vivo. **a** Time-course analysis of sTNFRII-MSC migrated to the spleen of CIA mice after transplantation. Detection of EGFP (green) expression and immunofluorescence staining for anti-human CD105 (red) were conducted in the spleen at 1, 3, 7, and 14 days after sTNFRII-MSC implantation. Scale bars: 200 μm. **b** The number of GFP^+^CD105^+^ cells was counted in the spleen on each of these days after sTNFRII-MSC implantation (n = 5). **c** Comparison of the numbers of retained GFP^+^CD105^+^ sTNFRII-MSC and EGFP-MSC in the spleen of CIA mice at 14 days after implantation. Scale bars: 200 μm. **d** Analysis of the numbers of GFP^+^CD105^+^ cells in the spleen of CIA mice at 14 days after implantation (n = 5). **e** Time-course analysis of sTNFRII-MSC migrated to the ankle joints of CIA mice after transplantation. Detection of EGFP (green) expression and immunofluorescence staining for anti-human CD105 (red) were conducted in the ankle joints at 1, 3, 7, and 14 days after sTNFRII-MSC implantation (n = 5). Scale bars: 200 μm. **f** Laser confocal was used to detect apoptosis of sTNFRII-MSC migrated into spleen of CIA mice at 14 days after implantation. The expression of cleaved caspase 3 (purple) was determined, and GFP (green) was used to locate migrated sTNFRII-MSC and EGFP-MSC. Scale bars: 25 μm. **g** Laser confocal was used to detect apoptosis of sTNFRII-MSC migrated into spleen of CIA mice at 14 days after implantation. The expression of LC3B-II (red) was determined, and GFP (green) was used to locate migrated sTNFRII-MSC and EGFP-MSC. Scale bars: 25 μm. Data are presented as the mean ± SD values. *p < 0.05, **p < 0.01

Previous studies demonstrated that systematically administered MSCs preferentially migrated to spleen tissue [37]. Therefore, we screened the spleen to verify the life-span of transplanted sTNFRII-MSC in vivo. CIA mice received an intravenous injection of 1 × 10^6^ of EGFP-MSC or sTNFRII-MSC and sacrificed on day 1, 3, 7 and 14 after cells infusion. Because the MSCs were isolated from human and modified to express EGFP, we used GFP and anti-human CD105 to co-locate sTNFRII-MSC in vivo. Sporadic GFP^+^CD105^+^ sTNFRII-MSC could be detected on day 1 following implantation and started to increase on day 3, then began to decreased on day 7 (Fig. 6a, b). GFP^+^CD105^+^ cells could still be detected until day 14. Moreover, we compared the abundance of GFP^+^CD105^+^ cells in spleen of CIA mice on day 14 after EGFP-MSC or sTNFRII-MSC injection. The results revealed that more GFP^+^CD105^+^ cells were detected in sTNFRII-MSC-infusion group (Fig. 6c, d). Ankle joints were also examined to validate if sTNFRII-MSC migrated to the inflamed region (Fig. 6e). GFP^+^CD105^+^ cells could be monitored in ankle joint on day 3 after infusion and till day 14. Based on the data above, we confirmed that systematically transplanted sTNFRII-MSC homed to inflamed region, and acted as immune-modulator and cartilage-protector locally. In addition, the life-span of sTNFRII-MSC is longer than that of EGFP-MSC, which partly explains its stronger effect in alleviating mice CIA.

### sTNFRII-Fc modification reduces apoptosis/autophagy in MSCs after implantation

Previously, we found that the life-span of sTNFRII-MSC is longer in vivo, thus we speculated that this is due to the occurrence of apoptosis and autophagy in MSCs induced by inflammatory environment. Therefore, we detected whether apoptosis and autophagy occurred in the MSCs migrated to the spleen following implantation, and the effect of sTNFRII-Fc modification. The laser confocal results showed that cleaved caspase 3 and LC3B-II could be detected in EGFP-MSC, yet almost no expression of cleaved caspase 3 (Fig. 6f) and LC3B-II (Fig. 6g) could be detected in sTNFRII-MSC. These results suggested that sTNFRII-Fc modification protected MSCs against apoptosis and autophagy after implantation.

### sTNFRII-Fc modification protects MSCs against apoptosis/autophagy induced by TNF-α and restores the immunosuppressive capacity of MSCs

**Fig. 7:**
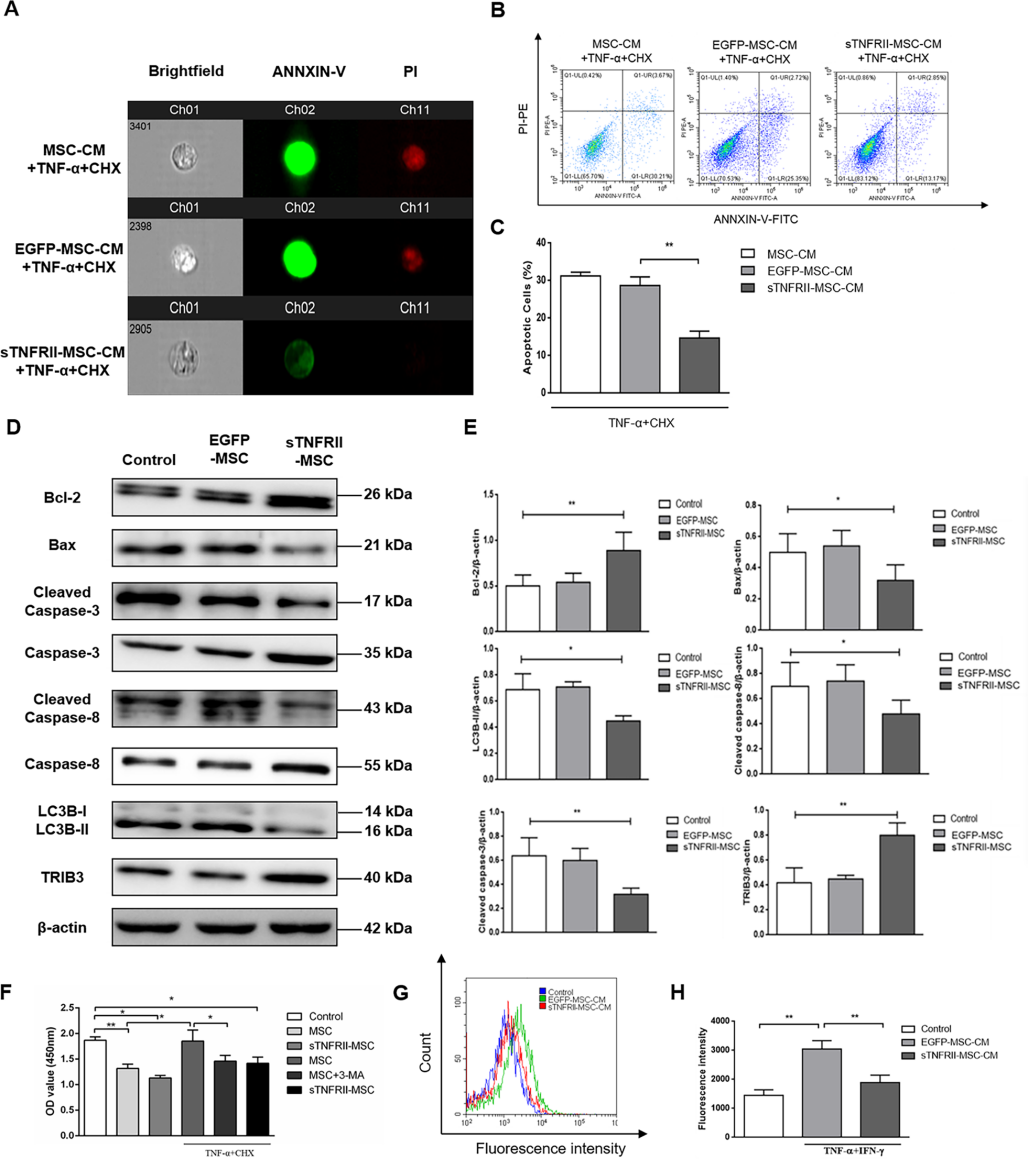
sTNFRII-Fc modification protects MSCs against apoptosis, autophagy and immunogenicity induced by TNF-α and restores the immunosuppressive capacity of MSCs. **a**, **b** Cells were incubated with MSC-CM, EGFP-MSC-CM or sTNFRII-MSC-CM in the presence of TNF-α + CHX as indicated, and stained with annexin V-FITC and PI-PE, and then analyzed by flow cytometry (**b**) and representative fluorescent images were collected by a imaging flow cytometer (**a**). **c** The percentage of apoptotic cells were analyzed (n = 5). **d** Cells treated with/without TNF-α + CHX, and the expression levels of Bcl-2, Bax, caspase-3, cleaved caspase-3, caspase-8, cleaved caspase-8, LC3B-II and TRIB3 were assessed by western blot. **e** Densitometry quantification of western blot from (**d**). The intensity of each was normalized to β-actin intensity (n = 3). **f** sTNFRII-Fc modification restores the immunosuppressive capacity of MSCs. Cells were treated as indicated, and the cell viability was measured by CCK-8 assay (n = 3). **g** The expression of HLA-DR on sTNFRII-MSC stimulated with TNF-α and IFN-γ was determined by flow cytometry. **h** Quantification of the fluorescence intensity from (**g**) (n = 3). Data are presented as the mean ± SD values. *p < 0.05, **p < 0.01

Many studies have illustrated that the internal inflammatory environment (especially TNF-α) is not conducive to MSCs’ immunoregulatory and tissue-regenerate capability by inducing apoptosis or autophagy [19, 20, 38]. We have proved that sTNFRII-MSC retained longer than MSCs without sTNFRII-Fc modification in CIA mice after systematic infusion, and since sTNFRII-MSC could binding and neutralizing TNF-α via secreting sTNFRII-Fc, we speculate whether the longer life-span of sTNFRII-MSC in vivo is due to the protective effect of sTNFRII-Fc modification against apoptosis and autophagy induced by TNF-α. Apoptotic parameters were analyzed by flow cytometry and western blot assay. Annexin V & PI staining revealed that the percentage of apoptotic MSCs (early apoptosis: Annexin V^+^PI^−^ + late apoptosis: Annexin V^+^PI^+^) were obviously enhanced in TNF-α/CHX treated MSCs in a time-dependent manner (Additional fi1e 1: Fig. 5A and 5B). The apoptosis-related proteins Bcl-2, Bax, caspase-3 and caspase-8 were then examined. After TNF-α/CHX treatment, the expression levels of Bcl-2, caspase-3 and caspase-8 decreased, while the expression levels of Bax, active form of caspase-3 (cleaved caspase-3) and active form of caspase-8 (cleaved caspase-8 increased) increased in a time-dependent manner for 6, 12, and 24 h (Additional fi1e 1: Fig. 5C and 5D). Moreover, the expression of autophagy-related proteins LC3B-II was increased, whereas the expression of tribbles pseudokinase 3 (TRIB3), an inhibitory molecule for autophagy was decreased in a time-dependent manner for 6, 12, and 24 h following TNF-α/CHX treatment, suggesting the apoptosis/autophagy-inducing effect of TNF-α on MSCs. Next, we evaluated whether sTNFRII-Fc modification could reduce the apoptosis/autophagy in MSCs induced by TNF-α. The flow cytometry results showed that sTNFRII-MSC-CM significantly decreased the percentage of apoptotic MSCs and reduced the intramembranous binding of Annexin V, as well as nuclear aggregation of PI (Fig. 7a–c). Meanwhile, the western blot results revealed that the expression of Bax, cleaved caspase-3, cleaved caspase-8 and LC3B-II decreased and the expression of Bcl-2, caspase-3, caspase-8 and TRIB3 increased in sTNFRII-MSC treated with TNF-α/CHX compared to the expression in EGFP-MSC (Fig. 7d, e). Additionally, we evaluated the effect of TNF-α-induced apoptosis/autophagy on immunosuppressive capability of MSCs on splenic CD4^+^ T cells from CIA mice. Figure 7f shows that TNF-α/CHX remarkably reversed the inhibitory effect of MSCs, but not sTNFRII-MSC on the proliferation of CD4^+^ T cells, and 3-MA (autophagy inhibitor) restored the CD4^+^ T cell-suppressive effect of MSCs. In summary, these data illustrated that sTNFRII-Fc modification protects MSCs against apoptosis/autophagy induced by TNF-α and restores the immunosuppressive capacity of MSCs.

### sTNFRII-Fc modification decreases immunogenicity of MSCs following inflammatory stimulation

Inflammation-induced immunogenicity is another obstacle hampering the therapeutic effect of MSCs [26]. TNF-α and IFN-γ synergistically elevated the HLA-DR level in MSCs. sTNFRII-MSC-CM, but not EGFP-MSC-CM or MSC-CM treatment reduced the HLA-DR level in MSCs (Fig. 7g, h).

### sTNFRII-Fc modification improves chondrogenic differentiation of MSCs following TNF-α stimulation

**Fig. 8:**
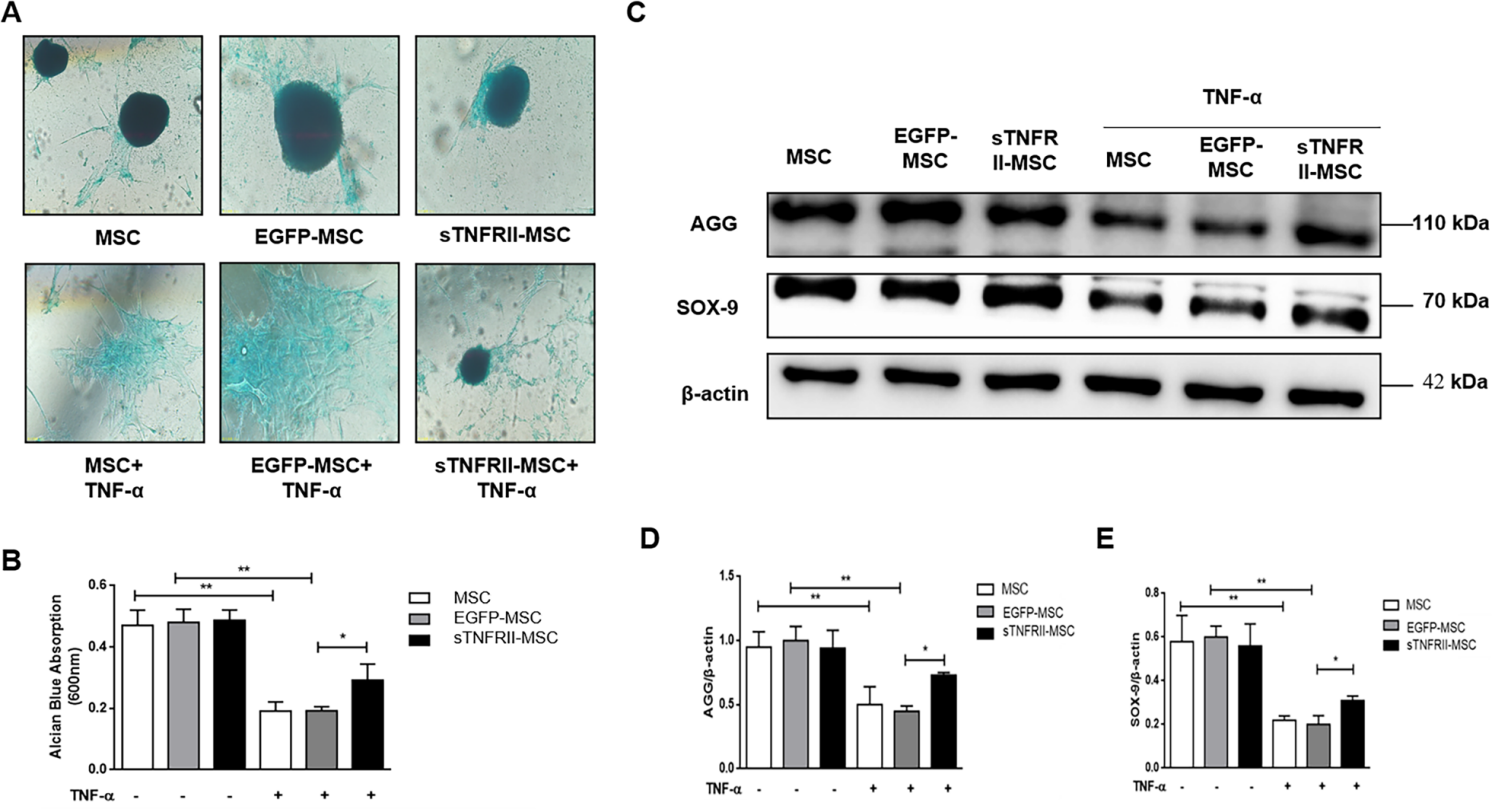
sTNFRII-Fc modification improves chondrogenic differentiation of MSCs following TNF-α stimulation. **a** Cells were treated with TNF-α, followed by assaying for chondrogenic differentiation. Alcian Blue staining was used to evaluate the chondrogenic capacity of sTNFRII-MSC. **b** The absorbance of eluent from (**a**) was determined at 600 nm for semiquantitation (n = 5). **c** Cells treated with/without TNF-α, and the expression levels of AGG and SOX-9 were assessed by western blot. **d**, **e** Densitometry quantification of western blot from (**c**). The intensity of each was normalized to β-actin intensity (n = 3). Data are presented as the mean ± SD values. *p < 0.05, **p < 0.01

In addition to the known inhibitory effect of TNF-α has on chondrogenesis, we tested the effect of sTNFRII-Fc modification on MSCs chondrocytic differentiation in the presence of TNF-α. Alcian blue staining revealed fewer cartilage spheres and lighter staining in all TNF-α treatment group, whereas sTNFRII-Fc modification partly counteracted this effect of TNF-α (Fig. 8a, b). Similarly, western bolt results showed that TNF-α inhibited the expression of AGG and Sox-9, which were chondrogenesis-associated proteins, in MSCs. As expected, higher levels of AGG and Sox-9 were observed in sTNFRII-MSC compared with EGFP-MSC or MSCs in the presence of TNF-α (Fig. 8c, d). The above data support that blockade of TNF-α is critical for cartilage regeneration.

## Discussion

Plenty of studies have proved the crucial role of TNF-α on RA pathogenesis, and TNF-α blockage using TNF-α inhibitors is currently the most effective therapeutic strategy for refractory RA [3, 12]. With the in-depth understanding of TNF-α inhibitors, certain possible safety risks are gradually discovered, including serious infection [11], malignancy [39] and immunogenicity [14]. Therefore, vectors have been explored to target the biologics to affected areas, such as synthetic polymers, viral/non-viral vectors and cells. However, application of synthetic polymer rose the concern on immunogenicity [40], and viral/non-viral vectors exerted potential risk associated with insertional mutagenesis [41]. Cell-based vectors, particularly MSCs, have been proved to be a promising vehicle for sustained delivery of bioactive factors in vivo [42]. With respect to the usage of MSCs-based vectors for gene and drug delivery, there are several preponderances. First, MSCs are immune-evasive, which enables implantation across major histocompatibility barriers [26]; second, MSCs exhibit rapid self-renewal with minimal senescence through multiple passages, providing possibilities for the in vitro expansion and subsequent application in clinic [42, 43]; third, MSCs can be easily isolated from various tissues, such as adipose, bone marrow and umbilical cord, and among which UC-MSCs have stronger immunotolerance-inducing ability as well as fewer ethical disputes than bone marrow-derived MSCs (BM-MSCs) (golden standard) [44]; fourth, MSCs have fewer chaos in the case of the insertion of transgenes via viral delivery. Genetic modification do not alter the intrinsic immune-modulatory and tissue-repairing capacity of MSCs, which endows adjunctively multi-targeted property of this vector [13, 45]; fifth, MSCs possess the innate targeting capacity owing to high homing to inflammatory and injured tissues [18, 46]. This characteristic allows accurate delivery of medicative molecules to affected areas. In this study, human UC-MSCs were served as carries, and genetically engineered to produce sTNFRII-Fc. UC-MSCs still maintained their intrinsic immunophenotyping, tri-lineage differentiation ability as well as immune modulating capability after sTNFRII-Fc modification. Following systemic transplantation, sTNFRII-MSC migrated to hyperinflammatory spleen and ankle joints tissues of CIA mice, indicating the targeted therapeutic effects of sTNFRII-MSC. In addition, no rejection reactions were observed during sTNFRII-MSC intervention, despite this xenogeneic implantation of human MSCs into mice. All these evidences supported the excellence of UC-MSCs-based vectors to delivery bioactive molecules for treating autoimmune diseases, such as RA.

Despite the aforesaid advantages of MSCs-based vehicle, low survival rate and impairment of implanted MSCs in vivo impeded the popularization of MSCs therapy [47, 48]. As a living cell intervention strategy, MSCs transplantation was sensitive to environmental associated apoptosis, such as high glucose [49], hypoxia [50], oxidative stress [51] and serum deprivation [52]. In recent studies, researchers have highlighted the negative effects of in vivo inflammatory microenvironment on MSCs, as MSCs were usually applied to treating inflammation and immune related diseases, like RA. Studies have illustrated that MSCs are defective, and the immunomodulation and differentiation functions of resident BM-MSCs are in standby, and even skewed toward pro-inflammatory phenotype in RA inflammatory condition [53]. Citrullinated fibrinogen exposure impaired the immunosuppressive effect of BM-MSCs by inhibiting the secretion of indoleamine 2,3-dioxygenase [54]. It was recently shown that BM-MSCs isolated from RA patients were lack of A20, which is responsible for the more IL-6 secretion [55]. In addition to the resident MSCs, similarly, implanted MSCs also sustained the pressure from the inflammatory environment after systemic infusion, which might partially explain the ineffectiveness of MSCs-based therapy in the treatment of RA [21, 22]. Inflammatory environment, especially TNF-α, notably mediated the impairment of MSCs via inducing apoptosis [19]. Correspondingly, we found that the number of MSCs was significantly fewer than that of sTNFRII-MSC in spleen of CIA mice, which was due to the occurrence of apoptosis induced by TNF-α. sTNFRII-MSC modification remarkably protected MSCs against TNF-α-induced apoptosis both in vivo and vitro by neutralizing TNF-α. Autophagy induced by TNF-α is another adverse factor affecting MSCs’ effects on autoimmune disease [20], and our data suggested the anti- autophagy function of sTNFRII-MSC. Additionally, MSCs have long been reported to be immune-evasive. However, inflammation (particularly TNF-α and IFN-γ) exposure increased the immunogenicity of MSCs by elevating major histocompatibility complex (MHC)-II expression, leading to the immune detection of these cells [26, 56, 57]. As expected, lower HLA-DR (MHC-II molecules) levels were observed in sTNFRII-MSC than un-modified MSCs after stimulated with TNF-α and IFN-γ. Furthermore, it has been proved that upregulation of VCAM-1 and ICAM-1 is essential for the immunosuppressive function of MSCs because it enables adhesion of T cells [58], whereas on the other hand, elevated VCAM-1 expression is associated with cytotoxic lysis of MSCs by alloantigen-specific cytotoxic T cells [57]. In our study, TNF-α notably upregulated ICAM-1 and VCAM-1 levels in MSCs, while both sTNFRII-MSC-CM and sTNFRII-Fc modification reduced the abundance of those two molecules in MSCs. In summary, our results indicated that sTNFRII-Fc modification protects human UC-MSCs against apoptosis, autophagy and immunogenicity induced by TNF-α, and thus enhances their efficacy in alleviating inflammatory and immune related diseases.

Subsequently, the therapeutic effects of sTNFRII-MSC were evaluated in mice CIA, which exhibits similar histological, pathological, and immunological profiles to human RA [30]. Results showed that the systemic transplantation of sTNFRII-MSC could significantly restore the body weight, reduce the pathologic scores of ankle joints and spleens, reduce the thymus/spleen index, and inhibit collagen II-specific CD4^+^ T cell proliferation. In addition, sTNFRII-MSC showed a stronger effect in inhibiting the AI, SJC and pathological bone erosion in CIA mice than MSCs and Etanercept. The better therapeutic outcomes of sTNFRII-MSC implantation could be explain by the protective effect of sTNFRII-Fc modification against apoptosis, autophagy and immunogenicity induced by TNF-α as described above.

Alteration of T cell subsets is often associated with the pathogenesis of RA. CD4^+^ T cells are highly malleable, and can differentiate into pathogenic T cells subsets such as Th1, Th17 and Tfh, instead of anti-inflammatory T cells subsets such as Th2 and Treg under the stimulation of RA's inflammatory microenvironment [59]. We tested whether the anti-arthritic effect of sTNFRII-MSC is related to modulation of T cell subsets. sTNFRII-MSC implantation reduced the percentage of Th1, Th17 and Tfh, while restored Th2 and Treg frequency in spleen of CIA mice. Associated pro-inflammatory cytokines (TNF-α, IFN-γ and IL-17) were decreased, whereas anti-inflammatory cytokines (IL-4 and IL-10) were elevated in sera of CIA mice after sTNFRII-MSC intervention. It is worth noting that sTNFRII-MSC exhibited stronger effect on inhibiting Th17 and inducing Treg than MSCs and Etanercept. In addition, B cells promote the occurrence and development of RA mainly through secreting autoantibodies, which causes abnormal immune response and tissue damage. At present, there are few studies concerning the effects of MSCs on B cells, mainly focused on inhibiting B cell autoantibody secretion, and promoting Breg generation [60, 61]. Our results showed that sTNFRII-MSC decreased the percentage of splenic plasma cells of CIA mice, and the elevation of Breg was only observed in sTNFRII-MSC treated mice. Moreover, levels of IgG2a and anti-CII were notably decreased in sera of CIA mice following sTNFRII-MSC infusion. Altogether, these findings suggested that sTNFRII-MSC can alleviated CIA in mice by modulating T and B cell functions, resulting in the upregulation of anti-inflammatory cytokines and the downregulation of proinflammatory cytokines as well as autoantibodies. Further, sTNFRII-MSC showed more promising therapeutic potential for CIA than MSCs and Etanercept.

Cartilage and bone destruction are major features of RA. There are three main causes of joint damage in rheumatoid arthritis. First, c-Fos, p53 signaling pathways are overactivated in FLS with the stimulation of inflammatory mediators (such as TNF-α and IL-1β), resulting in the enhanced proliferation and secretion of tissue degrading enzyme. Then, FLS infiltration occurred by degrading cartilage matrix [8, 62]. Second, inflammatory cytokines mediated the imbalance between RANKL and OPG in FLS, which is closely related to the osteoclast differentiation and subsequent bone erosion. Third, the inflammatory environment can lead to the disorder of synthesis-metabolism balance in chondrocytes, promoting the production of tissue degrading enzyme (specially MMP-13 and ADAMTS-5), mediating the degradation of cartilage matrix from the inside [8]. In this study, we evaluated the joint protective effect of sTNFRII-MSC. The data demonstrated that systemically implanted sTNFRII-MSC migrated to affected joints and significantly alleviated the pathologic change of CIA joints. In regard to mechanism, sTNFRII-MSC restored the MMP-13/TIMP-1 and RANKL/OPG ratio, and downregulated ADAMTS-5 levels both in chondrocytes and FLS. Moreover, sTNFRII-MSC exhibited stronger protective effect on cartilage matrix. As we expected, sTNFRII-MSC administration showed better joint-protective effects than MSCs and Etanercept.

**Fig. 9:**
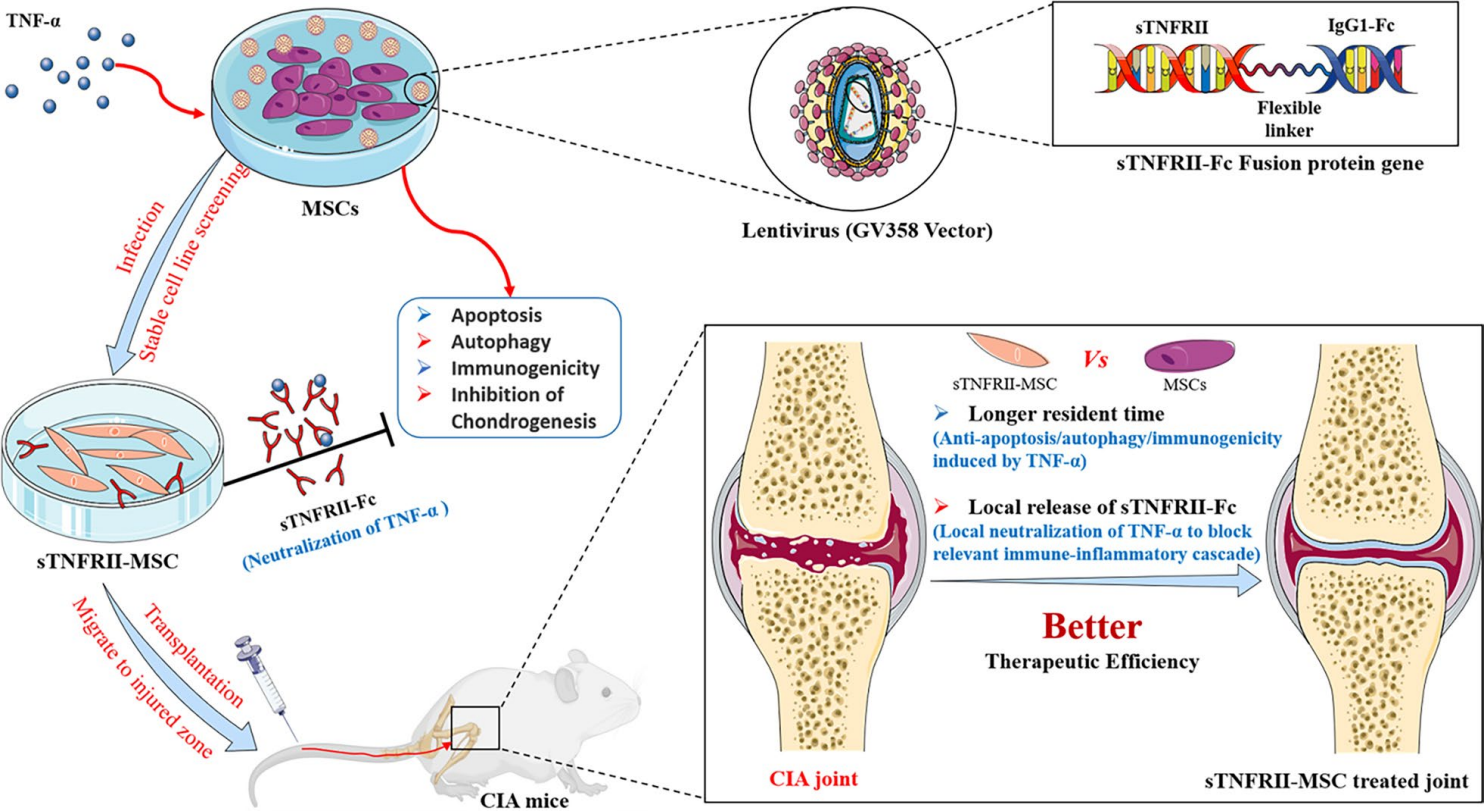
Possible mechanism through which sTNFRII-MSC exert better therapeutic efficiency in alleviating the development and severity of CIA in mice. sTNFRII-Fc modification protects MSCs against apoptosis, autophagy and immunogenicity induced by inflammatory environment (TNF-α), in addition to releasing sTNFRII-Fc neutralizing TNF-α to block relevant immune-inflammation cascade

Taken together, to our knowledge, our study provides the first evidence that sTNFRII-Fc modification protects human UC-MSCs against apoptosis/autophagy induced by TNF-α and enhances their efficacy in alleviating inflammatory arthritis. (Fig. 9) sTNFRII-MSC therefore provide a potential therapeutic measures and experimental basis for clinical treatment of autoimmune diseases such as RA.

## Conclusion

In this work, we constructed a multi-target cellular immunotherapy, which MSCs was used as a vector to deliver sTNFRII-Fc locally as well as exert their own immune-modulatory and tissue-repairing capacity, which provided new therapeutic measures and experimental basis for clinical treatment of autoimmune diseases such as RA.

## Supplementary Information

**Additional file 1**. **Fig. 1**. DNA sequence analysis of *sTNFRII-Fc* gene. (A) DNA sequence analysis of *homo sTNFRII*. (B) DNA sequence analysis of Fc domain of human IgG1. (C) Blast of *sTNFRII-Fc* with PrimeSTAR® HS DNA polymerase. (D) Representative pictures of sTNFRII-Fc gene. DNA Ladder (from top to bottom, 5 kb, 3 kb, 2 kb, 1.5 kb, 1 kb, 750 bp, 500 bp, 250 bp, 100 bp). sTNFRII-Fc gene was indicated by a red arrow; **Fig. 2.** The transfection efficiency of UC-MSCs was verified by flow cytometry (n = 5); **Fig. 3.** The gating strategies for flow cytometry detecting T- and B-cell subsets in spleen of CIA mice. (A) Th1: CD4^+^IFN-γ^+^. (B) Th2: CD4^+^IL-4^+^. (C) Th17: CD4^+^IL-17^+^. (D) Treg: CD4^+^CD25^+^Foxp3^+^. (E) Tfh: CD4^+^CXCR5^+^PD-1^+^. (F) plasma cells: CD19^−^CD138^+^. (G) Breg: CD19^+^IL-10^+^; **Fig. 4.** Transplantation of sTNFRII-MSC regulates the production of matrix degrading enzyme in ankle synovium of CIA mice. (A) Immunohistochemical staining of ADAMTS-5, MMP-13 and TIMP-1 in synovium of ankle joints from normal, CIA mice and mice treated with sTNFRII-MSC. The positive staining for ADAMTS-5 (B), MMP-13 (C) and TIMP-1 (D) was averaged. (E) The ratio of MMP-13/TIMP-1 in synovium of ankle joints from CIA mice was analyzed; **Fig. 5.** TNF-α + CHX induces apoptosis and autophagy in MSCs. MSCs were treated with TNF-α + CHX for 6, 12, and 24 h. (A) Time-course analysis of apoptosis of MSCs by Annexin V-FITC and PI-PE staining, and analyzed by flow cytometry. (B) The percentage of apoptotic cells were calculated in (A). (C) The expression levels of Bcl-2, Bax, caspase-3, cleaved caspase-3, caspase-8, cleaved caspase-8, LC3B-II and TRIB3 were assessed by western blot. (D) Densitometry quantification of western blot from (C). The intensity of each was normalized to β-actin intensity.

## Data Availability

All data generated or analyzed during this study are included in this published article.

## Abbreviations

AGG: Aggrecan
ADAMTS: A disintegrin and metalloproteinase with thrombospondin motifs
Breg: Regulatory B cells
BSA: Bovine serum albumin
cCII: Chicken collagen II
CIA: Collagen-induced arthritis
CM: Conditioned medium
ConA: Concanavalin A
FBS: Fetal bovine serum
HLA-DR: Human leukocyte antigen DR
ICAM: Intercellular adhesion molecule
IDO: Indoleamine-2:3 –dioxygenase
IFN: Interferon
IL: Interleukin
LPS: Lipopolysaccharide
MHC: Major histocompatibility complex
MMP: Matrix metalloproteinase
MSCs: Mesenchymal stem cells
OPG: Osteoprotegerin
PBS: Phosphate buffered saline
RA: Rheumatoid arthritis
RANKL: Receptor activator of nuclear factor κB ligand
sTNFRII: Soluble TNF receptor II
Tfh: Follicular helper T cells
Th: Helper T cells
Treg: Regulatory T cells
TNF: Tumor necrosis factor
TRIB: Tribbles pseudokinase
VCAM: Vascular cell adhesion molecule
