# Supplementary material for: sTNFRII-Fc modification protects human UC-MSCs against apoptosis/autophagy induced by TNF-α and enhances their efficacy in alleviating inflammatory arthritis
Source: Stem Cell Res Ther. 2021 Oct 9;12:535. doi: 10.1186/s13287-021-02602-4 (PMC8502322; doi:10.1186/s13287-021-02602-4)
Supplement: Supplementary file 1 — Additional file 1. Fig. 1. DNA sequence analysis of sTNFRII-Fc gene. (A) DNA sequence analysis of homo sTNFRII. (B) DNA sequence analysis of Fc domain of human IgG1. (C) Blast of sTNFRII-Fc with PrimeSTAR® HS DNA polymerase. (D) Representative pictures of sTNFRII-Fc gene. DNA Ladder (from top to bottom, 5 kb, 3 kb, 2 kb, 1.5 kb, 1 kb, 750 bp, 500 bp, 250 bp, 100 bp). sTNFRII-Fc gene was indicated by a red arrow; Fig. 2. The transfection efficiency of UC-MSCs was verified by flow cytometry (n = 5); Fig. 3. The gating strategies for flow cytometry detecting T- and B-cell subsets in spleen of CIA mice. (A) Th1: CD4+IFN-γ+. (B) Th2: CD4+IL-4+. (C) Th17: CD4+IL-17+. (D) Treg: CD4+CD25+Foxp3+. (E) Tfh: CD4+CXCR5+PD-1+. (F) plasma cells: CD19−CD138+. (G) Breg: CD19+IL-10+; Fig. 4. Transplantation of sTNFRII-MSC regulates the production of matrix degrading enzyme in ankle synovium of CIA mice. (A) Immunohistochemical staining of ADAMTS-5, MMP-13 and TIMP-1 in synovium of ankle joints from normal, CIA mice and mice treated with sTNFRII-MSC. The positive staining for ADAMTS-5 (B), MMP-13 (C) and TIMP-1 (D) was averaged. (E) The ratio of MMP-13/TIMP-1 in synovium of ankle joints from CIA mice was analyzed; Fig. 5. TNF-α + CHX induces apoptosis and autophagy in MSCs. MSCs were treated with TNF-α + CHX for 6, 12, and 24 h. (A) Time-course analysis of apoptosis of MSCs by Annexin V-FITC and PI-PE staining, and analyzed by flow cytometry. (B) The percentage of apoptotic cells were calculated in (A). (C) The expression levels of Bcl-2, Bax, caspase-3, cleaved caspase-3, caspase-8, cleaved caspase-8, LC3B-II and TRIB3 were assessed by western blot. (D) Densitometry quantification of western blot from (C). The intensity of each was normalized to β-actin intensity. [file 13287_2021_2602_MOESM1_ESM.pdf]

Supplementary Figure 1

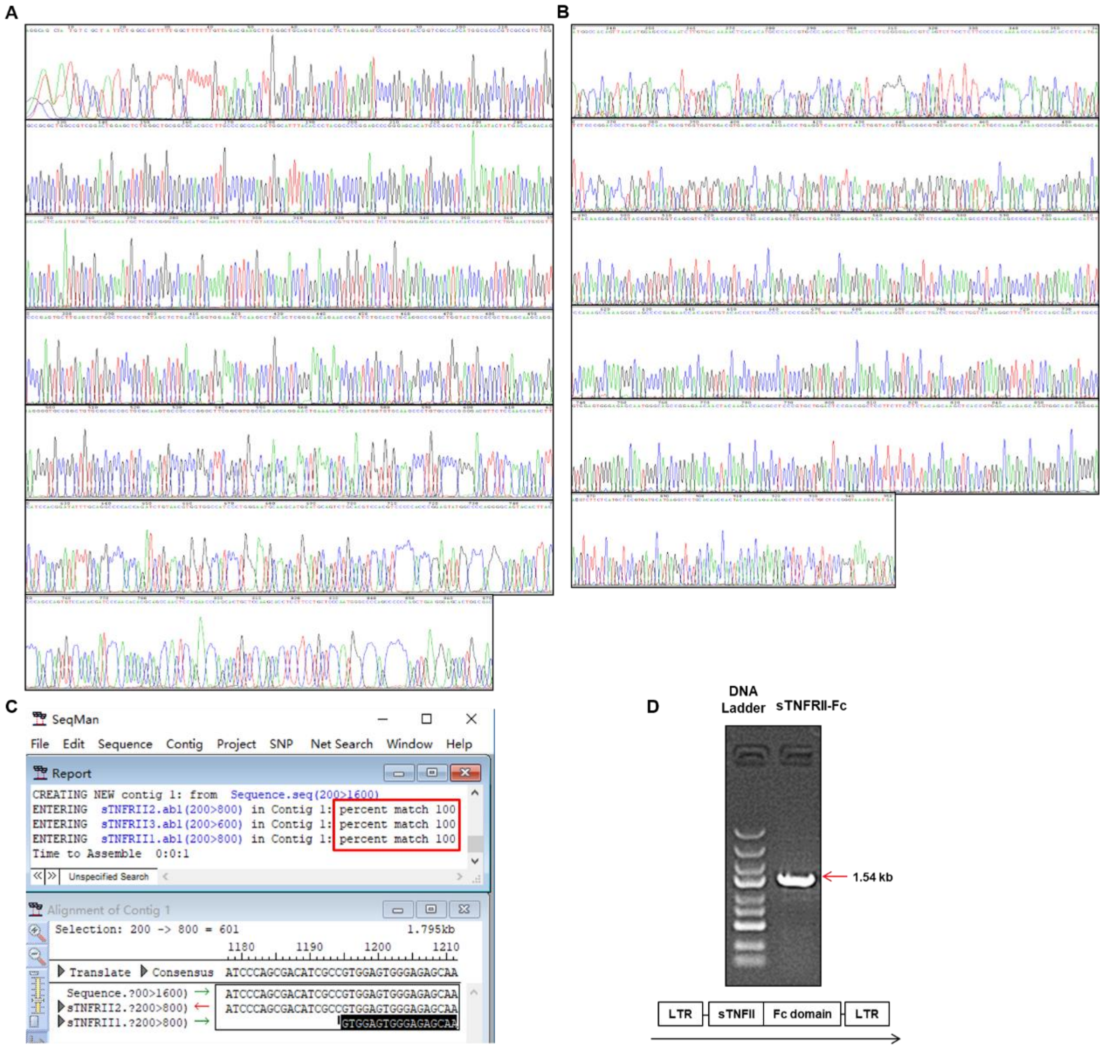

Supplementary Figure. 1 DNA sequence analysis of *sTNFRII-Fc* gene. (A) DNA sequence analysis of *homo sTNFRII*. (B) DNA sequence analysis of Fc domain of human IgG1. (C) Blast of *sTNFRII-Fc* with PrimeSTAR® HS DNA polymerase. (D) Representative pictures of sTNFRII-Fc gene. DNA Ladder (from top to bottom, 5 kb, 3 kb, 2 kb, 1.5 kb, 1 kb, 750 bp, 500 bp, 250 bp, 100 bp). sTNFR II-Fc gene was indicated by a red arrow.

Supplementary Figure 2

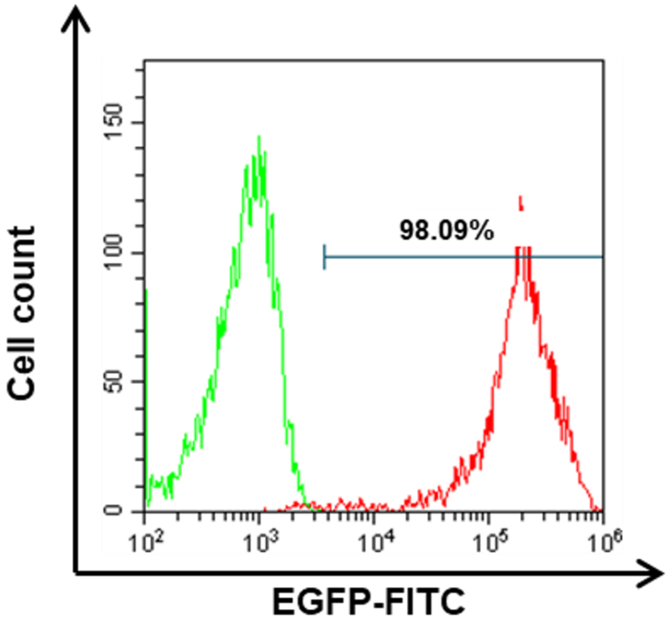

Supplementary Figure. 2 The transfection efficiency of UC-MSCs was verified by flow cytometry (n = 5).

Supplementary Figure 3

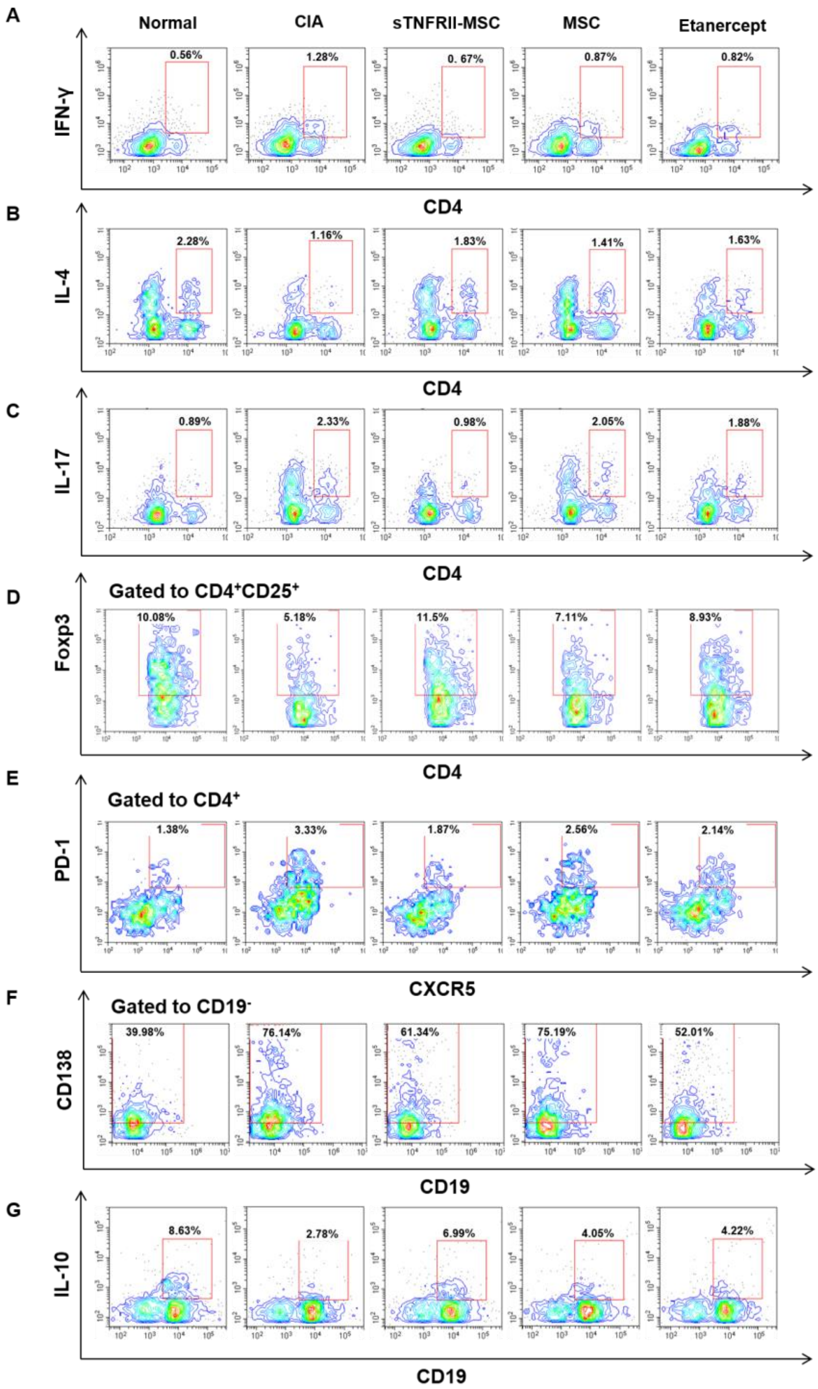

Supplementary Figure. 3 The gating strategies for flow cytometry detecting T- and B-cell subsets in spleen of CIA mice. (A) Th1: $CD4^{+}IFN\text{-}\gamma^{+}$. (B) Th2: $CD4^{+}IL\text{-}4^{+}$. (C) Th17: $CD4^{+}IL\text{-}17^{+}$. (D) Treg: $CD4^{+}CD25^{+}Foxp3^{+}$. (E) Tfh: $CD4^{+}CXCR5^{+}PD\text{-}1^{+}$. (F) plasma cells: $CD19^{-}CD138^{+}$. (G) Breg: $CD19^{+}IL\text{-}10^{+}$.

Supplementary Figure 4

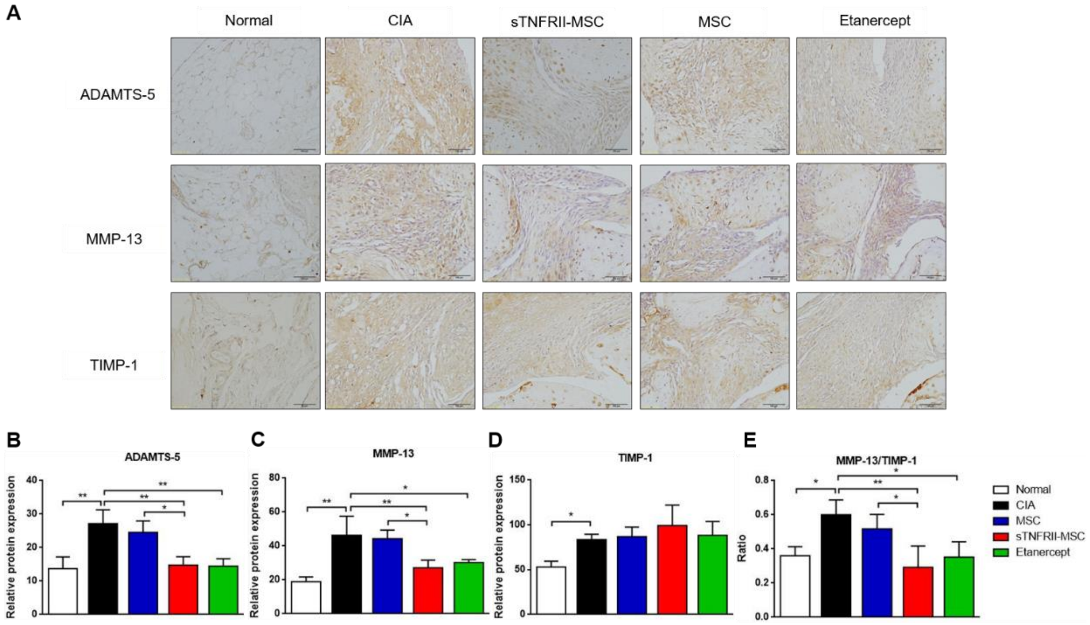

Supplementary Figure. 4 Transplantation of sTNFRII-MSC regulates the production of matrix degrading enzyme in ankle synovium of CIA mice. (A) Immunohistochemical staining of ADAMTS-5, MMP-13 and TIMP-1 in synovium of ankle joints from normal, CIA mice and mice treated with sTNFRII-MSC. Scale bars: 100 μm. Average optical density were analyzed individually in three different areas (n = 5). The positive staining for ADAMTS-5 (B), MMP-13 (C) and TIMP-1 (D) was averaged. (E) The ratio of MMP-13/TIMP-1 in synovium of ankle joints from CIA mice was analyzed. Data are presented as the mean ± SD values. $^{*}p < 0.05$, $^{**}p < 0.01$.

Supplementary Figure 5

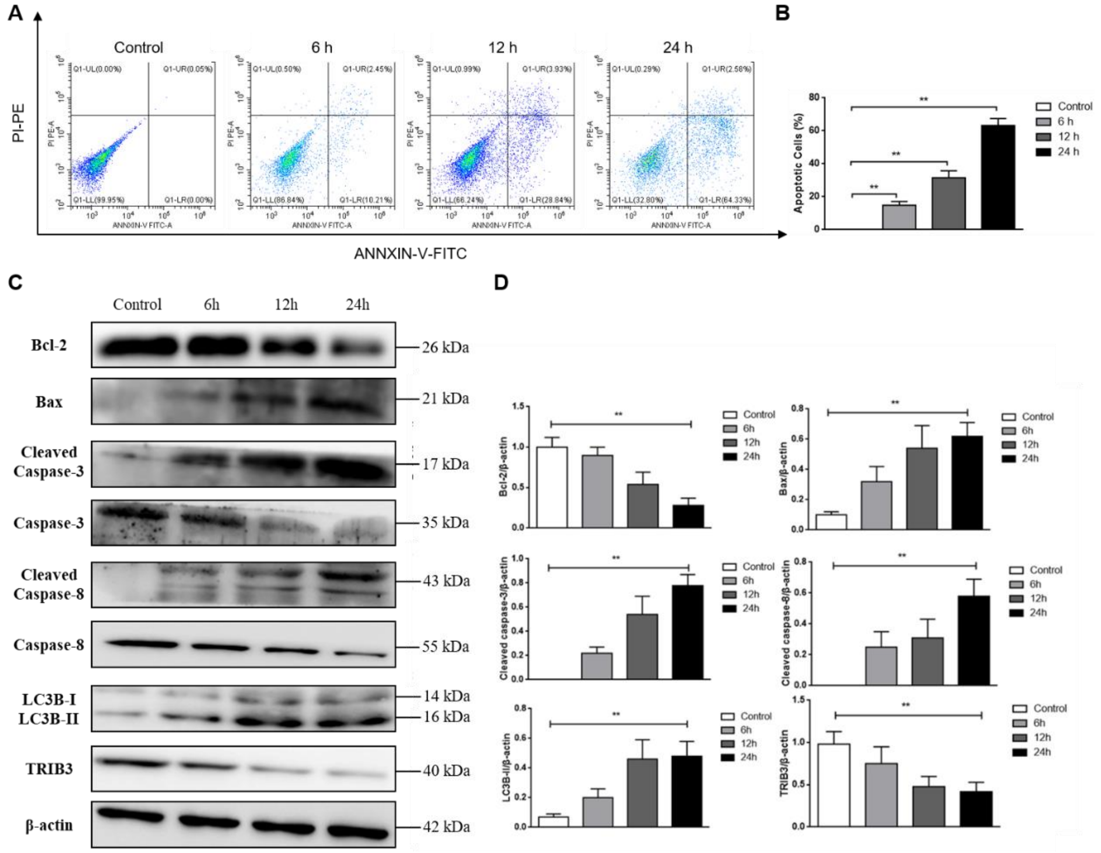

Supplementary Figure. 5 TNF-α+CHX induces apoptosis and autophagy in MSCs. MSCs were treated with TNF-α+CHX for 6, 12, and 24 h. (A) Time-course analysis of apoptosis of MSCs by Annexin V-FITC and PI-PE staining, and analyzed by flow cytometry. (B) The percentage of apoptotic cells were calculated in (A). (C) The expression levels of Bcl-2, Bax, caspase-3, cleaved caspase-3, caspase-8, cleaved caspase-8, LC3B-II and TRIB3 were assessed by western blot. (D) Densitometry quantification of western blot from (C). The intensity of each was normalized to β-actin intensity (n = 3). Data are presented as the mean ± SD values. $^{**}p < 0.01$.
